# Synergistic Advances in Additive Manufacturing and Surface Engineering for Polymeric Biomedical Devices

**DOI:** 10.1021/acspolymersau.5c00102

**Published:** 2025-11-07

**Authors:** Wei Juene Chong, Antonella Sola, Yuncang Li, Paul F.A. Wright, Cuie Wen

**Affiliations:** † Centre for Additive Manufacturing, School of Engineering, 5376 RMIT University, Melbourne, Victoria 3001, Australia; ‡ Department of Sciences and Methods for Engineering (DISMI), University of Modena and Reggio Emilia, Via Amendola 2, Reggio Emilia 42122, Italy; § School of Health and Biomedical Sciences, RMIT University, Bundoora, Victoria 3083, Australia

**Keywords:** polymer, additive manufacturing, surface functionalization, surface modification, bioactive coating, biomimetic coating, microfluidics, drug delivery, tissue regeneration, bone tissue engineering

## Abstract

Additive manufacturing (AM) of polymeric materials is rapidly transforming the biomedical field by enabling the fabrication of patient-specific, anatomically complex structures with precise control over internal architecture. Polymers are especially attractive for AM of biomedical devices due to their cost-effectiveness, abundance, low density, and tunable mechanical and degradation properties, supporting diverse applications in soft and hard tissue engineering, microfluidics, and drug delivery. However, many medical-grade polymers interact poorly with mammalian cells and tissues due to the lack of bioactive surface functional groups, which can hinder their performance in biomedical applications that rely on cell-material interactions such as tissue regeneration. This review systematically surveys physical, chemical, and biomimetic surface modification techniques for AM-compatible medical polymers to improve biomedical applications and targeted functionalities. While much attention has been paid in the literature to surface modification in bone tissue engineering, functional coatings incorporating bioactive molecules and nanoparticles further provide antibacterial, anti-inflammatory, and pro-regenerative functions. A major emphasis of this review is the synergy between AM and surface engineering, enabling simultaneous optimization of internal architecture and surface bioactivity—capabilities fundamentally unattainable by conventional manufacturing techniques. Finally, challenges such as sterilization compatibility and long-term stability of surface modifications are discussed as key to clinical translation.

## Graphical abstract

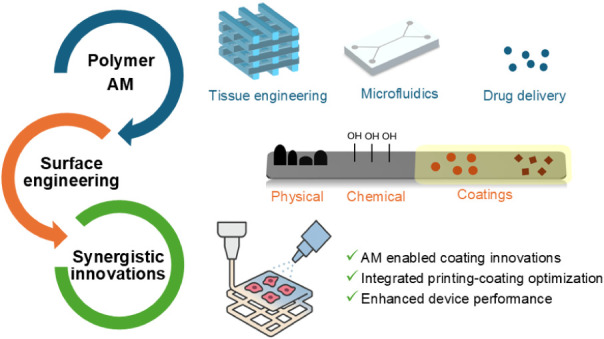

## 1 Introduction

Additive manufacturing (AM) is revolutionizing the healthcare industry by enabling the rapid and cost-effective fabrication of complex, bespoke biomedical devices.^1,2^ Unlike traditional subtractive processes (e.g., solvent casting and molding) that remove material from a bulk form, AM technologies build three-dimensional (3D) objects layer-by-layer, enabling the creation of intricate geometries^3^ (Figure 1). The general workflow entails the design of digital models which are created and then converted into file formats compatible with the printer.^3^ This approach is especially beneficial for the production of patient-specific devices, as it allows the translation of 3D scanned data, such as those obtained from computer tomography (CT) imaging, into digital models that can be processed into formats compatible with the relevant AM technologies to produce biomedical devices tailored to an individual’s unique anatomy.^4,5^

**Figure 1:**
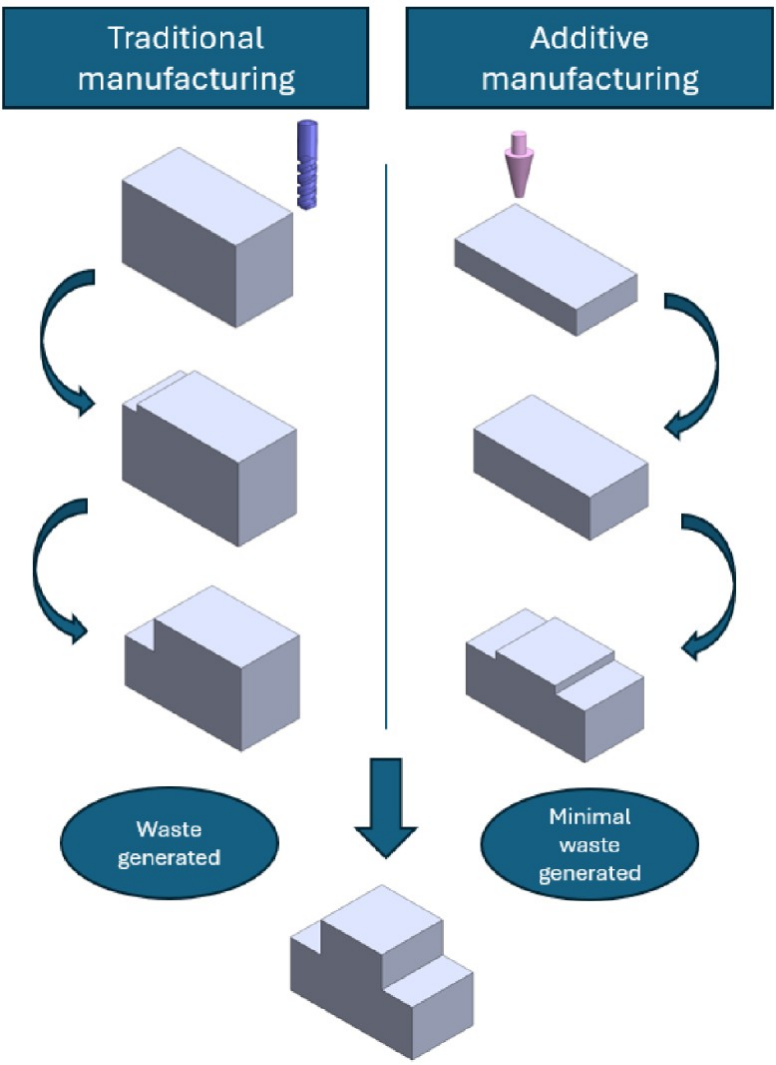
Comparison between traditional and additive manufacturing processes.

Among the various materials, AM-compatible polymeric materials, particularly Food and Drug Administration (FDA)-approved synthetic polymers for biological use such as polylactic acid (PLA), polycaprolactone (PCL), and polyether ether ketone (PEEK), are increasingly favored due to their widespread availability, affordability, and ease of processing.^6^ Their popularity is further driven by their highly tunable mechanical and degradation properties, which can be tailored to meet specific physiological requirements across a broad range of biomedical applications.^7^

Despite these advantages, most polymeric materials are inherently bioinert, that is, they interact minimally with surrounding mammalian tissues,^8^ which limits their effective use in biomedical contexts.^9^ However, it is important to note that this “bioinertness” does not prevent bacterial attachment on polymeric surfaces, since bacterial adhesion occurs through mechanisms fundamentally different from those of mammalian cells,^10,11^ leaving them still susceptible to bacterial contamination.

To address these shortcomings, two primary strategies, namely bulk modification and surface engineering, can be employed.^12^ Bulk modification involves incorporating bioactive or functional fillers into the polymer matrix to improve various properties including antibacterial performance, anti-inflammatory response, and tissue regenerative potential. However, this approach often alters the physical and mechanical properties of the material, potentially compromising its structural integrity and processability.^12^ In contrast, surface engineering targets the surface of polymeric biomedical devices by modifying properties such as roughness, topography, wettability, and chemical functionalities.^13^ This approach often enhances biological interactions between the polymeric device and its surrounding biological environment while preserving essential bulk properties that dictate the polymer’s suitability for specific applications.^13,14^

To examine the various surface engineering techniques used for AM polymeric biomedical devices and assess their effectiveness, a systematic review was conducted based on the research questions: (1) *What surface engineering techniques are applied to AM polymeric devices, and (2) how do they enhance the performance of these polymeric devices in the biomedical field?*

It is noteworthy that the interest in surface engineering for AM polymeric biomedical devices has increased steadily since 2014, parallel to the expansion of the material extrusion (MEX) AM technique known as fused filament fabrication (FFF, a.k.a fused deposition modeling (FDM)) following the expiry of Stratasys’ patent in 2009.^15,16^ The detailed research methodology and the comprehensive statistical and demographic analyses of research activity—including publication trends (Figure S2), polymer AM technique employed (Figure S3), and polymer types investigated (Figure S4)—are presented in the Supporting Information. A demographic analysis of the included studies is also provided there to offer insights into global research trends which may help inform future research and foster international collaborations in the field (Figure S5).

The literature analysis further reveals that surface engineering techniques have been applied across a wide range of biomedical applications, with bone tissue engineering receiving the most attention. Emerging studies have also explored applications in vascular, adipose, cartilage, skin, and skeletal muscle tissue engineering, as well as in microfluidic platforms and drug delivery systems.^17–20^ Despite this diversity, many of the reported surface modification strategies rely on common underlying principles, which can be broadly categorized as physical, chemical, or biomimetic approaches.

Popular physical methods such as plasma treatment and laser ablation may alter surface topography and increase roughness, which is widely associated with enhanced bioactivity due to increased surface area available for protein adsorption and cell attachment.^12,21–23^ Chemical methods typically introduce bioactive functional groups to improve surface wettability and promote cellular interactions. For example, alkaline hydrolysis and polydopamine (PDA) functionalization introduce hydrophilic groups like carboxyl and hydroxyl, which enhance hydrophilicity while serving as versatile platforms for further immobilization of biomolecules or nanoparticles.^24–27^ On the other hand, biomimetic approaches aim to replicate the features of native extracellular matrix (ECM). For instance, in bone applications, biomimetic coatings often incorporate organic and inorganic components such as collagen and hydroxyapatite (HA) to provide biophysical cues for cell attachment and differentiation.^27–29^ In addition, growth factors or peptides can be incorporated to deliver biochemical signals that further stimulate cellular responses and promote tissue regeneration.^30–32^ Additionally, functional nanoparticles with intrinsic bioactivity are also frequently added to impart antibacterial, anti-inflammatory, or regenerative properties.^26,33–36^ Collectively, these strategies highlight the adaptability of surface engineering for tailoring AM polymeric devices to meet diverse biomedical demands.

Importantly, this review emphasizes the synergy between AM and surface engineering in enabling innovative biomedical solutions that are difficult to achieve through conventional manufacturing methods. This synergy arises from the ability to simultaneously control the bulk architecture (e.g., complex geometries and porosity) and surface properties through various surface modification approaches (e.g., physical, chemical, coatings) during fabrication, allowing the integration of manufacturing versatility with innovative surface engineering strategies across diverse biomedical applications including tissue engineering, microfluidics, and drug delivery (Figure 2).

**Figure 2:**
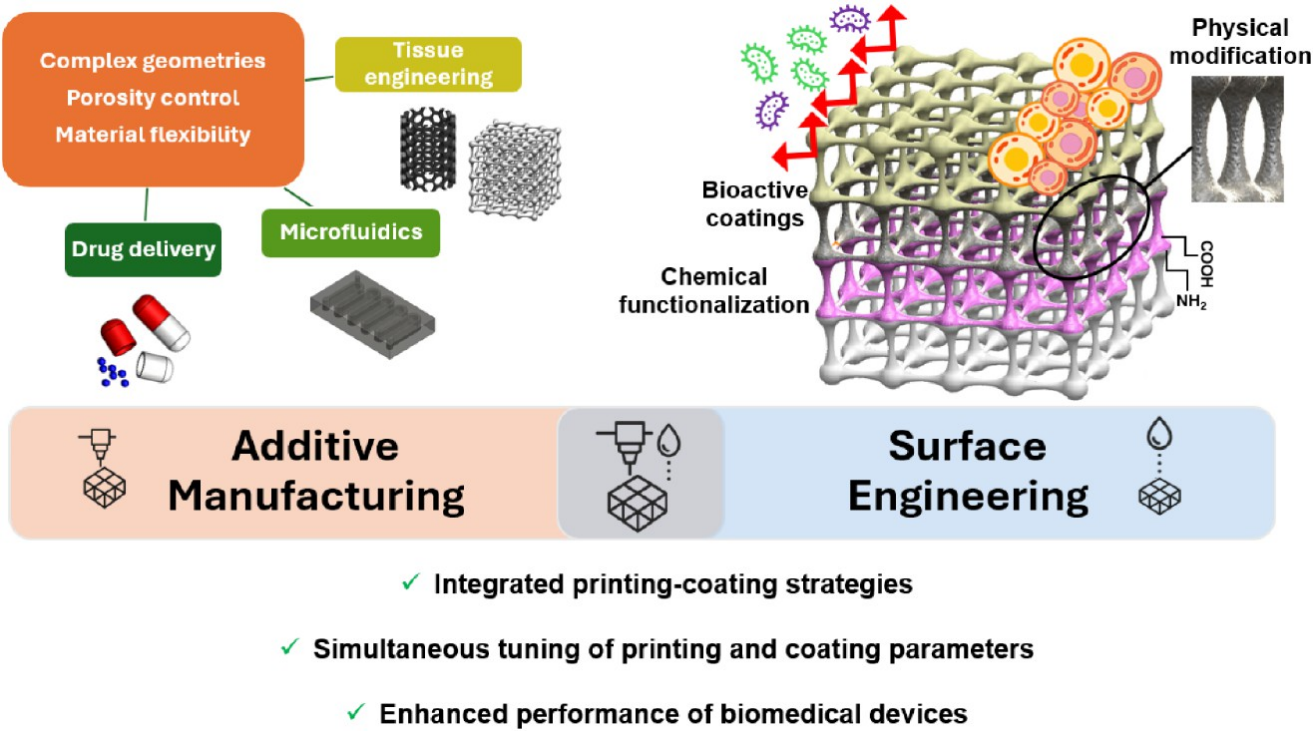
Illustration of the synergistic integration between AM and surface engineering, enabling enhanced performance of biomedical devices across diverse applications.

As an example, the infill, which defines the internal architecture of an AM part, can be tailored by adjusting its pattern type (e.g., triangle, honeycomb, rectilinear) and density (e.g., 10%, 50%, 100%) (Figure 3). By varying the infill density, controlled porosity can be introduced, which can in turn enhance coating absorption and consequently improves the mechanical performance of the printed part.^37^ Moreover, integrating surface treatment process directly within the layer-by-layer AM workflow can lead to more uniform coating coverage across complex geometries.^38^

**Figure 3:**
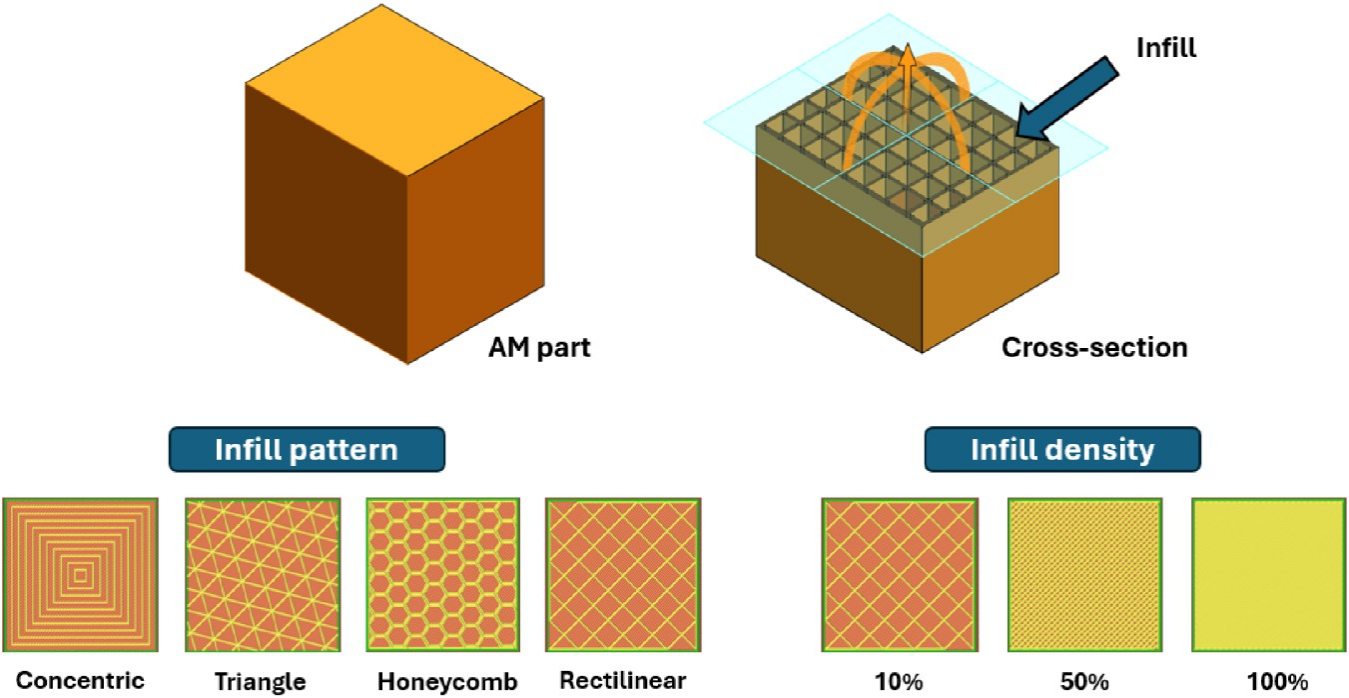
Illustration of the infill pattern and infill density in an AM part.

Given this synergy, surface-engineered AM polymeric devices hold significant promise for biomedical applications. This review aims to provide a comprehensive survey of the current surface engineering strategies applied to AM polymeric devices, with a goal of facilitating their clinical translation. To better guide future research in specific areas, the discussion is organized thematically based on the targeted biomedical application. The review also highlights current research gaps that must be addressed to unlock the potential of surface engineered AM polymeric devices in biomedical applications—most notably, the limited understanding of how mandatory sterilization processes affect device performance.

## 2 The Role of Polymer AM in Advancing Tissue Engineering, Microfluidics, and Drug Delivery Applications

One area where polymer-based AM has made significant contributions is tissue engineering. Polymers are well-suited for tissue engineering due to their cost-effectiveness, ease of processability, and importantly, their tailorable degradation rates to match the needs of the tissues being regenerated.^9,39^ This controllable degradability allows polymer-based tissue scaffolds to degrade gradually as natural tissue regenerates, eliminating the need for surgical removal.^40^ In addition to degradability, the mechanical properties of polymers are also highly tunable—ranging from soft, flexible thermoplastic polyurethane (TPU) to rigid materials like PEEK—making it possible to target a variety of natural tissues with differing mechanical characteristics.^36,41^ The tailorable degradation, combined with the ability of AM to form complex structures with precise porosity, makes AM polymeric scaffolds ideal for supporting cell growth and tissue regeneration.

It is important to note that, throughout this review, the term “scaffold” refers specifically to highly porous structures designed to promote cellular growth.^42^ This contrasts with implants or devices, which may not necessarily feature such porosity. While tissue scaffolds are generally considered to be biomedical devices, their porous architecture plays a key role in supporting tissue regeneration.^42–44^ The porosity supports 3D cell growth, which is necessary for shaping organs or tissues.^45^ Moreover, the porous architecture should be adjusted for any given applications, because the requirement for porosity varies depending on the targeted tissue type or functionality.^46^ For instance, bone tissue scaffolds require interconnected pores of specific sizes, typically ranging from 10 to 500 μm, to allow for cell seeding, nutrient exchange, and vascularization.^47^ A balance must be struck between porosity and mechanical strength to ensure that scaffolds provide sufficient structural support while promoting tissue growth.^48^

The advantages of AM in scaffold fabrication become particularly evident when considering the control it offers over pore architecture. AM enables the precise manipulation of key factors such as pore volume, pore size, and pore distribution, which are crucial aspects of tissue regeneration.^49,50^ Furthermore, AM allows for the creation of fully interconnected pore networks—something often unattainable with conventional fabrication methods like solvent casting and freeze-drying.^43,51,52^ This unparalleled control over porosity has driven the exploration in tissue engineering, which explains why many studies have focused on enhancing the surface properties of AM polymeric tissue scaffolds.

Beyond tissue engineering, surface engineering has also been applied to other AM polymeric biomedical devices, specifically drug delivery and microfluidic systems, though to a lesser extent. Advancement in polymer AM have also driven research aimed at improving the surface properties of these devices, as surface modifications are essential for optimizing their performances.^53,54^

Microfluidics involves the manipulation of fluids through micro- or nanoscale channels, offering precise control over small volumes and reducing sample consumption.^55^ This technology can be employed to analyze biomolecules from body fluids, serving as valuable *in vitro* diagnostic tools that aid in disease prevention, diagnosis, and treatment.^55–57^ With the growing demand for point-of-care diagnostics, Lab-on-a-Chip (LOC) devices are emerging as vital instruments that can be enabled by microfluidics.^58,59^ Traditionally, soft lithography was used to fabricate these devices.^60,61^ However, AM provides a more cost-effective alternative, eliminating the need for complex cleanroom setups required in soft lithography.^60^ In addition, polymers are increasingly favored over traditionally used materials such as silicon and glass for fabricating microfluidic devices due to their affordability, ease of production, and potential for large-scale manufacturing.^62^ This makes polymer AM an ideal solution for rapid prototyping of microfluidic devices,^63,64^ thus calling for novel surface engineering efforts aimed at improving the functionalities of these devices.

In the field of drug delivery, polymer AM is also achieving significant advancements in the development of drug delivery systems for administering pharmaceutical compounds to achieve therapeutic effects in the body.^65^ To minimize adverse effects while maximizing therapeutic efficacy, it is crucial for drugs to be delivered to specific target sites in a controlled and sustained manner.^65^ In this regard, polymers have long been used to incorporate bioactive agents due to their tailorable degradation rates, making them ideal for controlling the release of therapeutic agents at targeted sites.^66,67^ The flexibility of AM in customizing the design, size, shape, and porosity of drug delivery systems has further revolutionized this field, providing precise control over drug release kinetics and enabling personalized treatments that improve therapeutic outcomes and patient compliance.^68,69^

As research into AM polymeric scaffolds and biomedical devices progresses, there will likely be a growing emphasis on enhancing the surface properties of these biomedical devices. The bioinert nature of polymers presents challenges for many biomedical applications, making surface modifications crucial to improving their functionality and suitability for specific medical uses.^70^

## 3 Surface Engineering in AM Polymeric Biomedical Devices: A Thematic Overview

This review paper categorizes the literature into three primary themes based on the targeted applications of the surface-engineered AM polymeric devices examined in the reviewed studies. The first theme, which represents over half of the reviewed studies (see detailed search strategy in the Supporting Information; full study list in Table S1), focuses on bone-related tissue engineering applications. The second theme extends to other tissue engineering domains, including vascular, cartilage, adipose, dermal, and muscular tissues. The third theme encompasses applications beyond tissue engineering, such as drug delivery and microfluidic systems.

Before delving into specific surface engineering strategies, it is important to emphasize that surface pretreatments are commonly employed to activate the surface of AM-fabricated polymeric scaffolds and devices. These pretreatments serve as a crucial preparatory step, enabling the subsequent attachment of bioactive agents, functional materials, or coatings aimed at enhancing the overall performance of the device. Thus, the following section will explore various pretreatment techniques and their roles in preparing polymeric surfaces for functionalization.

### 3.1 Surface Pretreatment Strategies

Bioactive molecules such as growth factors (to directly stimulate biological responses) and functional materials (to improve biological or mechanical properties) are commonly employed to enhance the overall performance of AM polymeric devices.^71,72^ However, since most polymers are naturally bioinert, their surfaces are often pretreated to facilitate the attachment of bioactive and functional materials.^73^ Two of the most commonly used strategies for pretreating the surfaces of AM polymeric scaffolds and devices are alkaline hydrolysis and polydopamine (PDA) coating.

#### 3.1.1 Alkaline Hydrolysis

Alkaline hydrolysis using sodium hydroxide is widely used to activate the surfaces of polyesters, particularly PLA and PCL, to enable subsequent functionalization. This process cleaves ester bonds in an alkaline medium, generating hydrophilic carboxyl and hydroxyl groups on the surface. Additionally, the controlled degradation of polymer chains creates microscopic pits on the surface, which increase surface roughness and overall surface area,^74^ providing more attachment sites for bioactive molecules.^12,21^ Both the introduction of hydrophilic functional groups and the increased surface roughness are typically conducive to increasing a material’s affinity for biological interactions.^75–78^ Therefore, these surface properties will be revisited throughout this review as key contributors to bioactivity enhancement.

Interestingly, alkaline hydrolysis alone has been shown to ameliorate the bioactivity of AM polymer-matrix composites through the controlled degradation of the matrix. This mechanism was demonstrated, for example, by Backes et al.,^79^ for PCL/HA composite scaffolds. Alkaline hydrolysis gradually degraded the molecular chains of the PCL matrix, subsequently exposing the embedded HA on the surface of the composite, which were responsible for a significant bioactivity enhancement.^79^

Nonetheless, alkaline hydrolysis is still more frequently employed as a pretreatment strategy of polymer constructs in preparation for further surface functionalization.^25,27,34,80,81^ The functional groups introduced during alkaline hydrolysis, particularly carboxyl groups, provide essential sites for attaching bioactive or functional molecules. For instance, carboxyl groups facilitate the binding of sericin (silk protein),^25^ and other compounds such as polyethylenimine (PEI) acting as the substrate for citric acid (CA),^28,34^ which in turn mediates the deposition of bioactive materials like HA and functional agents like cerium oxide (CeO_2_) nanoparticles. Additionally, carboxyl groups have been utilized to facilitate the stable deposition of biomimetic hydrogel/calcium phosphate (CaP) coatings onto AM PLA scaffolds by enabling silane attachment as an intermediate layer.^80^

While alkaline hydrolysis is an effective method for surface activation and functionalization, it is important to carefully control the exposure time, as excessive degradation can compromise the mechanical properties of polymeric devices, especially scaffolds having fine architectures.^79,82^

#### 3.1.2 Polydopamine (PDA) Coating

PDA is widely recognized for its outstanding adhesive properties, enabling it to adhere to nearly all types of surfaces, regardless of the substrate’s chemistry.^83^ Its adhesive capabilities are often compared to those of mussels, owing to the presence of catechol and amine functional groups in dopamine, which closely resemble those found in 3,4-dihydroxyphenylalanine (DOPA)—a key component of the adhesive proteins secreted by mussels.^26,27,30,84,85^

Numerous strategies have been developed to immobilize bioactive molecules onto the surface of polymeric scaffolds. However, many of these techniques involve complex chemistry, which often introduces undesirable toxic components.^27^ Mussel-inspired PDA functionalization has been introduced as a simple, one-step approach to improving the attachment of bioactive and functional materials to the surfaces of AM polymeric devices. The abundance of highly reactive functional groups in PDA, including amine, imine, and catechol groups, allows it to effectively immobilize a wide range of bioactive molecules through covalent and noncovalent interactions.^86^ For instance, Teixeira et al.^27^ reported the ability of PDA to increase the binding efficiency of collagen Type I (COL I) to PLA scaffolds by up to 92%, which was attributed to covalent interactions between the functional groups of PDA and COL I. Furthermore, Seok et al.^85^ demonstrated that PDA coatings enabled the tunable deposition of graphene oxide (GO) onto PCL scaffolds through covalent interactions between GO’s epoxy groups and PDA’s catechol hydroxyl groups. This led to the controlled deposition of GO, which is essential to prevent adverse effects resulting from excessive GO loading.

As discussed, PDA contains highly reactive functional groups that promote biomolecular interactions while also being inherently hydrophilic. Consequently, even when applied independently, PDA coatings have demonstrated desirable biological responses such as osteogenic and angiogenic differentiation.^26,87^

Interestingly, aside from biological properties, Sharma et al.^37^ demonstrated the potential for PDA coatings to improve the mechanical properties of AM PLA bone plates. This was achieved by optimizing the parameters of printing (infill density, layer height, print speed) and PDA coating (immersion time, shaker speed, coating concentration), leading to an improvement of up to approximately 95% in tensile strength and 34% in flexural strength.^37^ The coordinated optimization of printing parameters and coating parameters was key to this success. For instance, lowering the infill density allowed for better PDA coating absorption due to the increased voids. To a certain extent, PDA absorption was even able to compensate for the usual reduction in tensile and flexural strengths associated with increased voids. Moreover, extending the coating immersion times also led to higher PDA concentrations.^37^ As a result, this enhanced the bonding between the PDA coating and the PLA bone plate, providing greater stability and strength, which helped the bone plates to withstand higher loads.^37^

### 3.2 Bone Tissue Engineering

Bone tissue engineering has emerged as one of the most extensively researched applications within surface engineering for AM polymeric biomedical devices. The first study in this domain was published in 2014, and continuous progress has been made through 2024.

Bone tissue engineering has been a leading area of tissue engineering due to the growing clinical need for bone regeneration therapies, especially in light of an aging population.^88^ As life expectancy increases, so does the prevalence of bone-related conditions such as fractures and defects, thus triggering the urgent need for scalable solutions in bone healing and regeneration.^89^

Traditional treatment methods, such as autografts (harvesting bone from the patients’ own body) and allografts (from a donor), present several limitations.^90^ Autografts are limited by donor site morbidity, limited availability, and high-cost of harvesting, whereas allografts carry risks of immune rejection and disease transmission, and may not be osteogenic.^90^ These challenges have spurred significant research efforts in bone tissue engineering, aiming to develop engineered scaffolds that promote bone regeneration while overcoming the limitations of conventional grafting techniques.

The success of a bone tissue scaffold depends on its ability to not only support cellular attachment, but also actively guide cellular behavior to facilitate bone formation.^91^ Aside from the critical role of porosity as mentioned in Section 2, an effective scaffold must be biocompatible and capable of mimicking the biochemical and biophysical cues in the native bone environment that are essential for bone regeneration.^92^ This has been achieved through surface engineering approaches which can be broadly categorized into three main strategies: (1) physical and chemical modifications, (2) incorporation of extracellular matrix (ECM)-mimicking components, and (3) the addition of functional materials with inherent functionalities.

In brief, physical and chemical modifications involve modifying surface properties such as roughness, hydrophilicity, and chemical groups to optimize cellular behavior,^93^ while the incorporation of ECM-mimicking components, such as growth factors and proteins that are bioactive, can directly stimulate cellular responses to enhance bone regeneration.^71,94,95^ Additionally, functional materials, while not directly mimicking ECM components, can modify surface properties or introduce chemical groups to improve biological responses, while also providing inherent functionalities such as antibacterial activity and inflammatory response modulation.^34,35^ These techniques are discussed further in the following sections.

#### 3.2.1 Physical and Chemical Surface Modification Techniques

This section explores the physical and chemical surface modification strategies utilized to enhance the bioactivity of AM polymeric devices by modifying surface properties such as topography, roughness, chemical composition, and wettability, all of which directly influence cellular behavior.^96,97^ Specifically, techniques like plasma treatment, laser treatment, and acetone immersion provide significant advantages by enabling the creation of nanoscale features that more accurately replicate the native bone architecture, at a level of precision that AM technologies alone often cannot achieve.^98^ Moreover, these methods offer the advantage of enhancing bioactivity while circumventing the complexities typically associated with the direct incorporation of bioactive components.^99^ The following section examines each of these surface modification techniques and their respective impacts on the bioactivity of AM polymeric devices.

##### 3.2.1.1 Plasma Treatment

Plasma treatment involves ionizing a gas in a vacuum chamber to form a plasma, which is typically a partially ionized substance consisting of positive charges, negative charges, and un-ionized neutral molecules.^100,101^ Plasma treatment can be categorized into hot, warm, and cold plasmas.^102^ Hot plasmas operate at extremely high bulk plasma temperatures often reaching thousands of degrees Celsius, where the electrons and ions are highly energetic.^102^ Warm plasmas have a lower bulk temperature than hot plasmas, yet they can still be too hot for treating polymeric materials.^102^ In contrast, cold plasmas such as Cold Atmospheric Plasma, a.k.a Low-Temperature Plasma, have high-energy electrons while maintaining a low overall plasma temperature.^102^ This makes them well-suited for treating heat-sensitive AM polymeric devices to mitigate thermal degradation.^103^

During plasma treatment, the applied energy breaks the chemical bonds within the polymer, generating free radicals.^104^ These radicals interact with the surrounding gas environment, producing new surface functional groups such as hydroxyl, carboxyl, carbonyl, and amine, which are highly hydrophilic and known to enhance the biological affinity of polymeric surfaces.^93,104^ Additionally, the bombardment of high-energy ions on the surface removes some material, creating nanoscale irregularities and increases surface roughness, providing additional binding sites for biomolecules, and finally enhancing the scaffold’s bioactivity.^12,21,22,105^ Collectively, the increase in hydrophilic bioactive functional groups and surface roughness has been shown to improve the bioactivity of AM PLA and PEEK scaffolds, promoting cell adhesion, metabolic activity, proliferation, and osteogenic differentiation.^23,98,106^ Moreover, due to this ability to improve the biological affinity of polymeric surfaces, plasma treatment can also be used as pretreatment to prepare the surfaces of AM polymeric scaffolds for further functionalization.^25,106,107^

Notably, the effect of plasma treatment on bioactivity enhancement is highly dependent on the type of gas utilized. For instance, Han et al.^24^ demonstrated that oxygen (O_2_) plasma outperforms argon (Ar) plasma in improving the bioactivity of AM PEEK discs due to the higher concentration of oxygen-containing functional groups, which promote the adhesion and differentiation of osteoblasts (bone-forming cells). Furthermore, these oxygen-containing functional groups are highly hydrophilic, leading to improved surface wettability compared to Ar-treated surfaces.^24^ Similarly, Mohsenimehr et al.^23^ showed that nitrogen (N_2_) plasma enhances bioactivity by introducing hydrophilic nitrogen-containing functional groups, such as amides, imides, and imines. Among the tested gas compositions (pure N_2_, 1:1 N_2_:O_2_, 1:1 N_2_:Hydrogen (H_2_), 1:1 N_2_:O_2_), 1:1 N_2_:O_2_ provided the greatest bioactivity enhancement.^23^ Although the 1:1 N_2_:H_2_ plasma provided the highest hydrophilicity, its bioactivity enhancement was less effective than that of 1:1 N_2_:O_2_ due to the reaction between nitrogen and hydrogen radicals, which reduced the availability of nitrogen radicals to bind with the polymer surface to form nitrogen-containing functional groups.^23^ Consequently, 1:1 N_2_:H_2_ plasma treatment was not as optimal as 1:1 N_2_:O_2_ plasma treatment, where a balance of hydrophilic oxygen and nitrogen groups promoted superior cell adhesion and proliferation.^23^

Beyond gas type, factors such as treatment time and power supply have also been shown to affect cellular response due to changes in surface roughness.^23,98^ Wang et al.^98^ highlighted that increasing treatment times (1, 3, and 5 min) significantly impacted bioactivity by increasing surface roughness. Furthermore, Mohsenimehr et al.^23^ demonstrated that increased power supply enhances surface roughness by intensifying etching. Both studies show that increased surface roughness led to improved scaffold bioactivity.^23,98^ This is because the nanoscale features created by these treatments closely resemble the natural ECM, which is known to positively influence cell adhesion.^23^ These nanofeatured surfaces enhance cell adhesion by improving integrin binding, resulting in better cell clustering and spreading, which, in turn, promoted osteoblast differentiation and bone formation.^98^ However, careful control of plasma treatment parameters is essential to achieve optimal surface modification, as excessive treatment time or power supply can lead to overetching or damage to the surface.

Typically, plasma treatments are applied as a separate step after the printing process. However, Liu et al.^38^ introduced a novel in-process plasma treatment approach, integrating plasma modification during printing using their in-house-developed plasma-assisted bioextrusion system (Figure 4).

**Figure 4:**
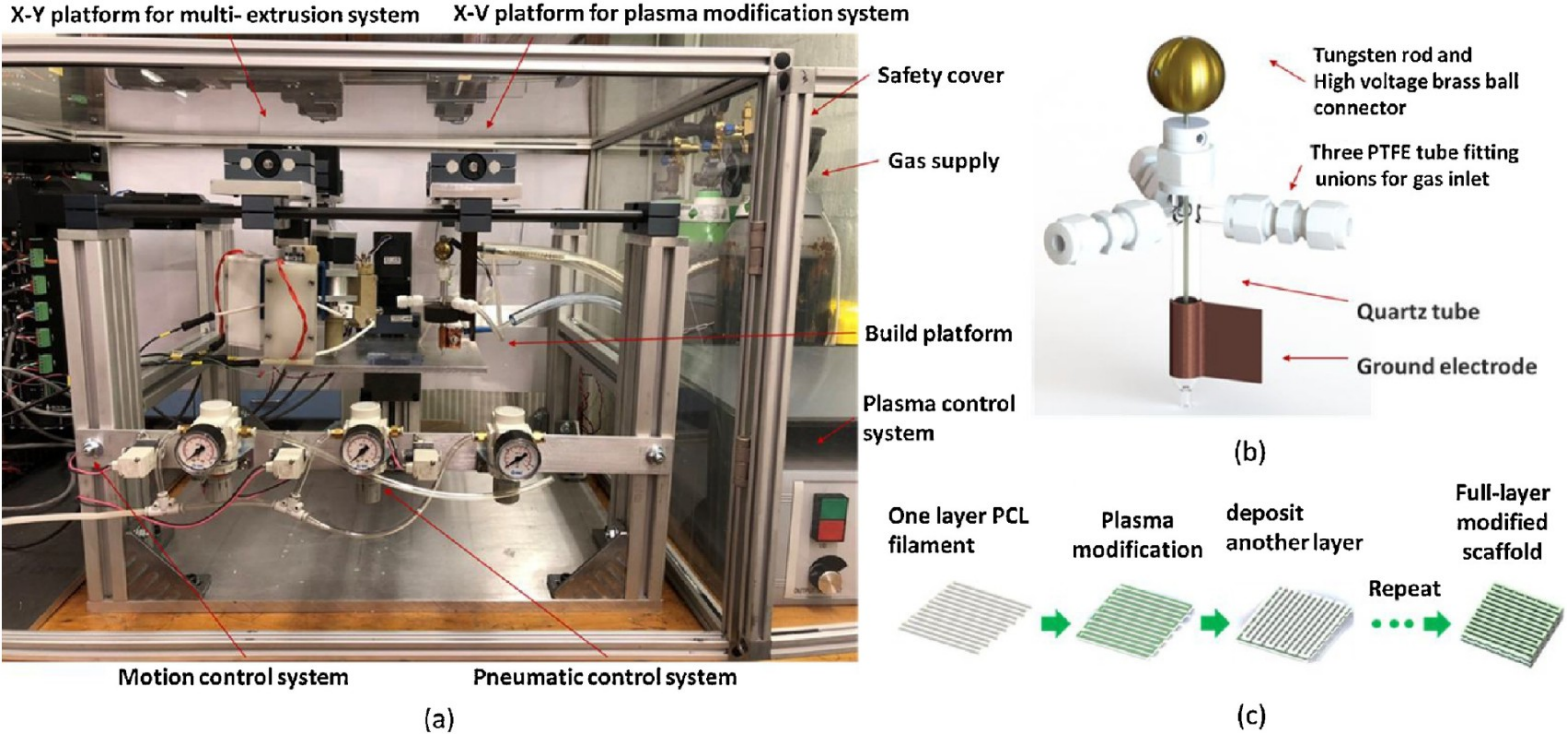
Plasma-assisted bioextrusion system developed by Liu et al.^38^ Reproduced with permission. Copyright 2018, Elsevier.

As shown in Figure 4, this method applies a layer-by-layer plasma treatment to the molten material during the printing process, enhancing gas flow effects on surface topography.^38^ The simultaneous modification during printing also improves the uniformity and homogeneity of plasma effects across the entire structure, representing a significant advancement in treating the surface of AM scaffolds.^38^

The relative simplicity of plasma treatment and its ability to enhance surface properties without altering the bulk properties make it a popular surface engineering technique for polymeric scaffolds and devices. However, this technology has a notable limitation—the nonpermanent nature of the hydrophilicity it induces.^82^ Plasma-treated surfaces tend to recover some of their hydrophobicity over time,^24,98^ a phenomenon known as hydrophobic recovery.^108^ This recovery occurs due to mechanisms such as segmental mobility of polymer chains, desorption of low-molecular-weight fragments, and reorientation and migration of polymer chains, where the extent of recovery is dependent on the polymer type and on the plasma treatment conditions.^108^ To mitigate this issue, studies have shown that storing plasma-treated samples in closed, dry environments and at temperatures below ambient can effectively delay this recovery.^23,98^

By stabilizing and prolonging its effects, plasma treatment can be a valuable technique for enhancing the bioactivity of polymeric scaffolds.

##### 3.2.1.2 Laser Treatment

Laser treatment is often employed to create patterns and textures on the surface of polymeric components.^109^ This technique involves focusing a laser beam onto the material’s surface to selectively heat and melt specific regions, enabling the development of micro- and nanostructures.^110^ Specifically, femtosecond laser treatment enables the creation of nanostructures with high precision, as they emit ultrashort pulses with durations in the femtosecond range (10^– 15^ s).^111^ Each laser pulse delivers an intense amount of energy in an extremely short time frame, rapidly evaporating material in the focal spot without significant heat dissipation into the surrounding area, making it a popular technique for texturing heat-sensitive polymeric devices, including scaffolds.^111^

Currently, the research on femtosecond laser treatment specifically aimed at improving the bioactivity of AM polymeric scaffolds and devices is still an emerging field. Notably, two studies by Filipov et al.^112,113^ explored the potential of femtosecond laser treatment in improving the bioactivity and antibacterial properties of AM PCL scaffolds. Two topographies—microchannels and microprotrusions—were fabricated by altering the number of laser pulses (Figure 5).^112^ Among the two, microchannels demonstrated superior performance, likely owing to their higher wettability and organized linear topography.^112^ The increased wettability facilitated better protein adsorption and osteoblast adhesion, while the linear structure of the microchannels promoted cell alignment and enhanced osteogenic differentiation.^112^ Importantly, the microstructures remained stable for up to 72 weeks, providing sufficient durability to support the initiation of tissue repair, which typically occurs between 5 to 15 days.^112^ However, it should be noted that this level of durability may exceed what is functionally necessary, prompting further investigation to determine whether such extended stability offers additional benefits or if material properties could be optimized for more efficient degradation timelines. Nonetheless, the durability of these microstructures is influenced by factors such as the polymer’s intrinsic degradation rate and topography size,^114–116^ necessitating case-by-case assessment.

**Figure 5:**
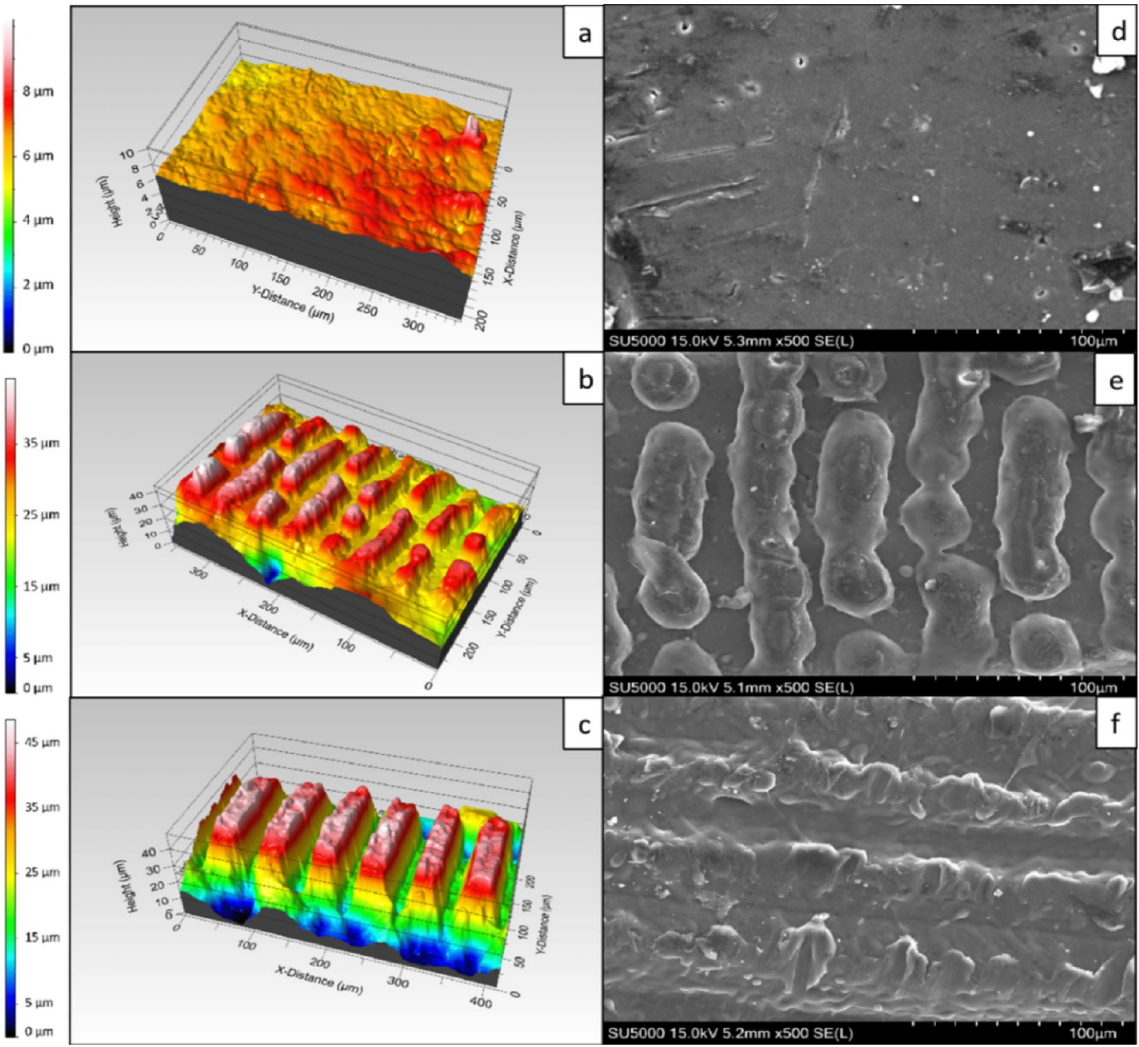
3D and scanning electron microscopy (SEM) profiles of PCL scaffolds irradiated with a femtosecond laser. (a,d) Control sample; and topographical features created with laser fluence of 0.08 J/cm^2^ and different numbers of applied pulses: (b,e) 2 pulses for producing microprotrusions, and (c,f) 10 pulses for microchannels. Reproduced from ref 112. Available under a CC-BY license. Copyright 2022.

In terms of antibacterial effects, microchannels more effectively impeded *Staphylococcus aureus* (*S. aureus*) biofilm formation compared to *Escherichia coli* (*E. coli*).^112^ The microchannel roughness, being smaller than the average size of bacterial cells, disrupted the adhesion of *S. aureus*.^112^ On the other hand, *E. coli* was less affected by mechanical disruption, due to the additional resistance provided by its thin peptidoglycan layer and outer membrane.^112^ Overall, Filipov et al.^112^ demonstrated the potential for the laser-induced microchannels to selectively disrupt bacterial cells due to their rigid walls, while sparing mammalian cells due to their larger size and flexible membranes.

Although the first study by Filipov et al.^112^ demonstrated the potential of using laser treatment for enhancing the bioactivity and antibacterial properties of AM PCL scaffolds, it is evident that the antibacterial properties, particularly against *E. coli*, were suboptimal.^112^ To address this, Filipov et al.^113^ subsequently introduced an additional modification by combining femtosecond laser treatment with atomic layer deposition (ALD) of zinc oxide (ZnO), aiming to enhance the antibacterial efficacy of the scaffold.

Given the low melting temperature of PCL (∼58 °C), the study primarily focused on overcoming the challenge of depositing ZnO at lower temperatures,^113^ well below the conventional ALD processing window of 130 to 180 °C.^117^ To prevent the thermal degradation of PCL, ZnO deposition was performed at 50 °C, with varying ALD cycles used to achieve coatings of increasing thickness on the laser-structured scaffolds. Under these conditions, Filipov et al.^113^ confirmed that ZnO nanolayers adhered uniformly to the scaffolds.

Notably, the laser-patterned surfaces exhibited enhanced ZnO adsorption, particularly at the bottoms of the microchannels, as compared to untreated surfaces. According to Filipov et al.,^113^ this preferential ZnO deposition could be attributed to the formation of reactive chemical groups and to an increase in surface energy during laser ablation. These localized changes created stronger adsorption sites, leading to higher ZnO accumulation in the patterned regions compared to untreated PCL. While no antibacterial studies were conducted in this work,^113^ the well-documented antibacterial properties of ZnO strongly suggest that this coating has significant potential to improve the scaffold’s antibacterial efficacy.^118,119^

##### 3.2.1.3 Acetone Immersion

Wang et al.^87^ employed acetone immersion to modify the surface topography of AM PCL scaffolds to improve their bioactivity. Although the authors have referred to their method as acetone vapor annealing, it is important to clarify that the scaffolds were actually immersed in pure acetone solution, rather than being exposed to acetone vapors. This distinction is crucial, as solvent vapor annealing typically involves exposure to solvent vapors only (such as acetone vapor), rather than immersion in liquid solvents.^120,121^ Therefore, this review has referred to their method as acetone immersion instead.

According to Wang et al.,^87^ acetone softens the amorphous domains of PCL. As acetone evaporates, the amorphous domains recrystallize onto the existing crystalline regions within the polymer, resulting in the formation of a hierarchical surface structure with microporosity and nanoscale roughness (Figure 6A).^87^

**Figure 6:**
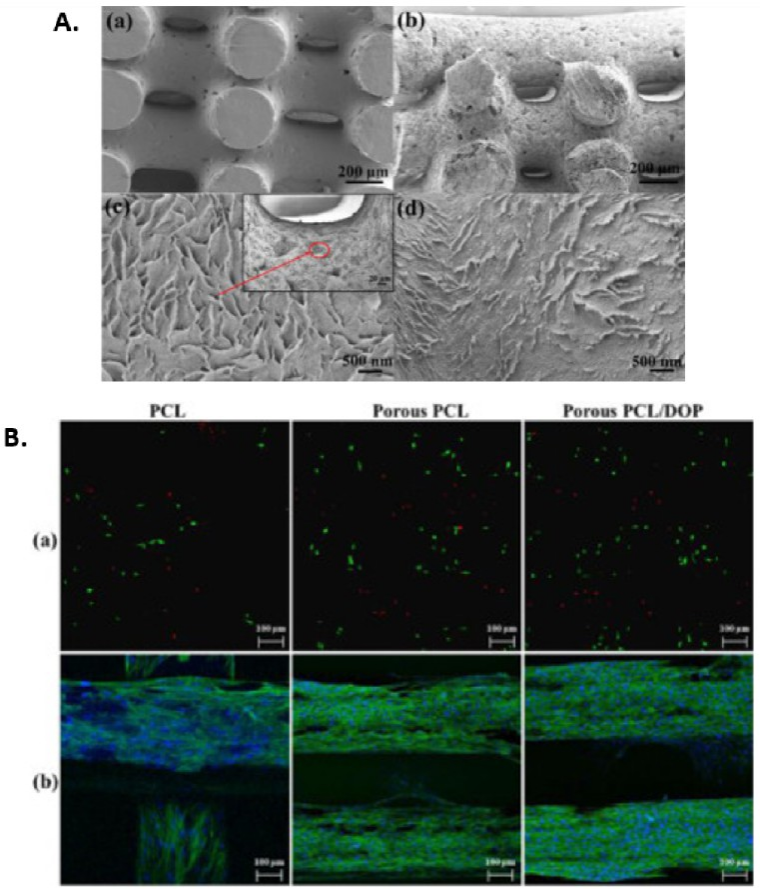
A) Structural morphologies of PCL scaffolds: (a) neat PCL; (b) porous PCL after acetone immersion (low magnification); (c) porous PCL after acetone immersion (high magnification); (d) PDA-coated porous PCL (PCL/DOP). B) Confocal microscope images (scale bar = 100 μm (200×)): (a) live/dead staining at 1 day after cell seeding (live cells in green and dead cells in red); (b) immunofluorescence staining after 14 days of cell culture (cell nuclei stain blue, and cell actin stain green). Reproduced with permission.^87^ Copyright 2020, Elsevier.

Notably, the increased surface roughness improved the scaffold’s bioactivity, as evidenced by the achievement of higher cell attachment and proliferation as compared to the untreated scaffolds.^87^ As shown in Figure 6B, the effectiveness of acetone immersion in enhancing scaffold biocompatibility was further amplified when combined with PDA coating, evidenced by extensive cell attachment, cell spreading and the formation of confluent cell sheet.^87^ In this synergistic approach, the modified surface created by acetone immersion serves as an optimal foundation for PDA coating adhesion, which greatly enhanced the scaffold’s biological performance.^87^ The role of PDA in augmenting scaffold bioactivity has been discussed in detail in the previous section (Section 3.1.2).

#### 3.2.2 Incorporation of ECM Mimicking Components

ECM is a naturally occurring substrate that provides structural support and serves as a reservoir for bioactive molecules, including proteins, growth factors, and cell signaling factors.^122^ These components are essential for facilitating the various cellular processes involved in tissue regeneration, including cell adhesion, migration, differentiation, and proliferation.^123^ The critical role of ECM in tissue regeneration, including bone tissues, has been well documented,^124^ with widespread research on engineered scaffolds that aim to mimic the native bone ECM environment that comprises both inorganic and organic components as further examined in the following sections.

##### 3.2.2.1 Inorganic Component

The inorganic component of bone ECM predominantly consists of CaP, primarily in the form of HA, which provides bones with strength and stiffness.^125^ Osteoblasts produce CaP crystals that are deposited within the collagen matrix, leading to bone mineralization (a.k.a. bone calcification).^125^ As the bone matures, these crystals grow and accumulate, strengthening the bone.^125^ To mimic the bone’s mineral composition, various CaP-based ceramic materials have been utilized for bone tissue engineering applications. Among them, HA, beta-tricalcium phosphate (β-TCP), biphasic calcium phosphate (BCP), and calcium-deficient HA have been considered useful to promote bone tissue growth.^126^

Studies on functionalized AM polymeric scaffolds have confirmed the osteoinductivity and osteoconductivity of calcium-deficient HA, as well as the osseointegrability of HA, which collectively enhance the scaffold’s bioactivity.^28,29^ For example, Jaidev et al.^28^ reported enhanced cell adhesion, proliferation, and stem cell osteogenesis in HA-coated AM PLA as compared to untreated PLA (Figure 7). Notably, they also compared hydrolyzed scaffolds and with and without HA coating, demonstrating that while hydrolysis improved bioactivity by increasing surface roughness and hydrophilicity, the subsequent HA coating further boosted bioactivity through the sustained release of calcium ions (Ca^2+^) into the cell culture medium.^28^ In particular, the release of Ca^2+^ has been shown to promote bone mineral formation and regulate the expression of osteogenic differentiation-related genes,^127^ thus, supporting the role of Ca^2+^ in stimulating bone cellular activity.

**Figure 7:**
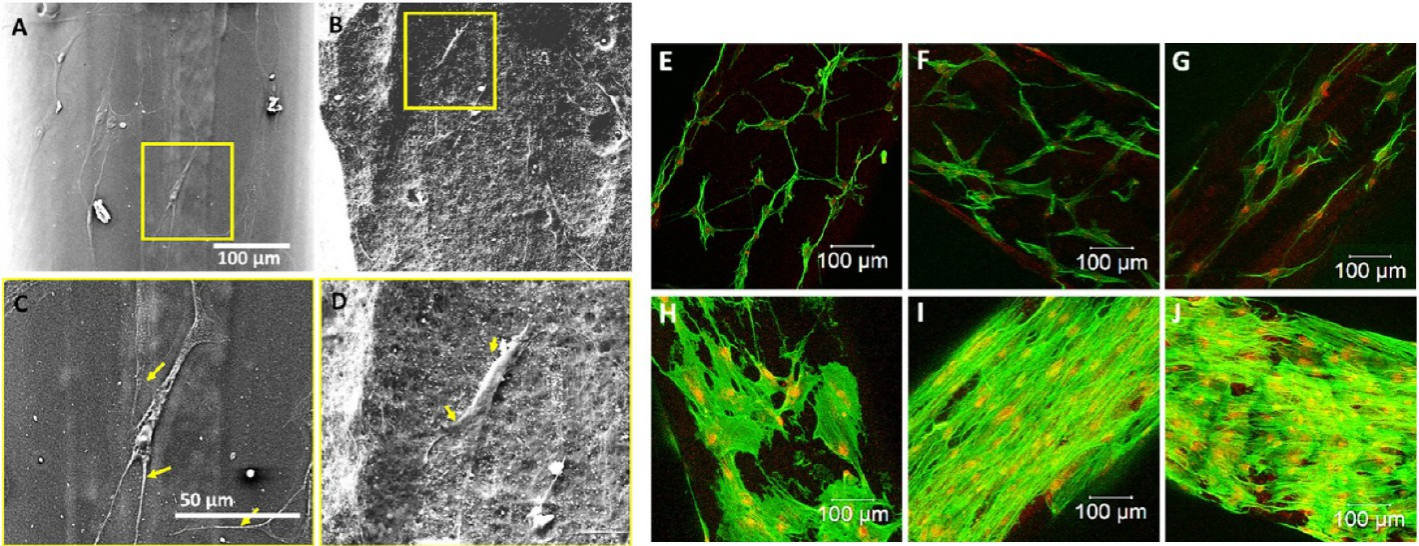
Response of hMSCs cultured on PLA and surface-functionalized 3D-printed scaffolds. [A–D] SEM images at low (A,B) and high (C,D) magnifications showing cell attachment on day 1 for PLA (A,C) and PLA-HaP (B,D). [E–J] Confocal microscopy images of fluorescently labeled cells on day 1 (E–G) and day 7 (H–J), showing F-actin (green) and nuclei (red) for PLA (E,H), hydrolyzed PLA (F,I), and HA-coated PLA (G,J). Adapted from ref 28. Available under a CC-BY-NC-ND license. Copyright 2018.

Moreover, Oladapo et al.^29^ demonstrated that HA induced the formation of apatite on AM PEEK scaffolds when immersed in simulated body fluid (SBF), suggesting the ability of the functionalized scaffold to support osseointegration. Typically, bone-bonding materials form an apatite layer upon implantation, and this aids their bonding with adjacent bone tissues.^128^ Since the SBF mimics the composition of human blood plasma, it is commonly used to test the ability of scaffolds to form apatite upon immersion as a form of bioactivity preassessment.^128^

Despite the benefits of HA, a single material often cannot achieve the desired bone-healing outcomes, and a combination of different bioactive materials is typically required for optimal results. For this reason, Menezes et al.^129^ coated AM poly­(butylene adipate-*co*-terephthalate) (PBAT) with HA in combination with either bioglass or gelatin, demonstrating that both combinations led to biocompatible coatings. However, bioglass was regarded as the key factor in promoting the proliferation of bone-forming cells, while HA mainly contributed to the enhancement in mechanical properties,^129^ due to the inherent mechanical resistance of CaP.^130^

Apart from investigating the biological effects of CaP-based coatings, a significant portion of research has focused on the methods for applying these coatings onto AM polymeric scaffolds. These methods include SBF immersion, cold-spraying, and the use of dental resin cement coating. A summary of these methods is outlined in Table 1.

**Table 1:** Methods Utilized to Coat AM Polymeric Scaffolds and Devices with CaP-Based Coatings

Method	Pretreatment	Matrix material	Description	Ref.
Cold spraying	None	PEEK scaffold	Compressed gas (nitrogen or helium) is used to accelerate HA powder and spray it onto the substrate’s surface at pressures ranging from 0.5 to 2.0 MPa. Upon impact, the powder undergoes physical deformation and adheres to the surface without melting or undergoing any chemical reactions, forming a layer of HA coating on the surface.	^131^
Dental resin coating	None	PA 12 disc	10-Methacryloyloxydecyl dihydrogen phosphate (10-MDP) dental resin cement was used to attach the HA powder to the substrate’s surface. The resin cement, which consists of two pastes, was mixed and applied to the substrate. After applying the uncured resin, the HA powder was spread over it. The resin holding the HA powder was then light-cured for 20 s to bond the HA powder securely to the substrate’s surface.	^132^
Dip coating	Alkaline hydrolysis, then APTES coating	PLA scaffold	Two solutions were prepared to coat the substrate with a hybrid CaP/hydrogel (either chitosan or gelatin). The first solution contained chitosan or gelatin mixed with calcium nitrate tetrahydrate and dissolved in 1% acetic acid, while the second solution contained disodium hydrogen phosphate and glutaraldehyde. The scaffolds were dipped into the first solution for 5 min, followed by immersion in the second solution for another 5 min. This dipping cycle was repeated 15 times, and the hybrid coating was completed by drying the samples overnight.	^80^
SBF immersion	Alkaline hydrolysis, followed by PEI conjugation and CA binding	PLA scaffold	The substrates are immersed in SBF, which mimics the ionic composition of human blood plasma. The surface functional groups, introduced during pretreatment, interact with Ca^2+^ ions in the SBF, forming Ca–polymer complexes that serve as nucleation sites.^133^ These sites attract phosphate (PO_4_^3 –^) ions, leading to the formation of crystal nodules.^133^ These nodules further react with additional ions to form CaP crystals.^133,134^	^28^
	Low-temperature ethylenediamine plasma treatment	PLA scaffold		^106^
	PDA coating	PCL scaffold		^30^

Among the methods outlined in Table 1, SBF immersion is the most widely used technique to coat AM polymeric surfaces with CaP. As previously mentioned, this method is also the standard approach for a preliminary assessment of a scaffold’s bioactivity by evaluating its ability to induce apatite formation. Both applications share the same underlying principle, as described in Table 1. However, neat polymeric surfaces typically lack functional moieties to promote interaction with SBF.^135^ Therefore, surface activation through pretreatments, such as PDA coating and alkaline hydrolysis, can be used to facilitate the deposition of CaP onto AM polymeric scaffolds during immersion in SBF. This is exemplified in the studies of Park et al.^30^ and Jaidev and Chatterjee,^28^ (cited in Section 3.1 as part of the pretreatment strategies).

Specifically, Park et al.^30^ showed that PDA coating enhances CaP deposition on PCL scaffolds by releasing hydrogen ions in SBF, leading to the local accumulation of bicarbonate and hydrogen phosphate ions, which in turn triggers the nucleation and growth of CaP. On the other hand, Jaidev and Chatterjee^28^ adopted a multilayer approach starting with alkaline hydrolysis, followed by PEI conjugation and treatment with CA, which facilitated the attachment of Ca^2+^ and phosphate ions (PO_4_^3 –^) from SBF, finally enabling HA precipitation.^28,80^

In contrast to the surface coating methods described above, Bradford et al.^106^ proposed a novel strategy, using low-temperature ethylenediamine plasma treatment to introduce reactive functional groups that serve as nucleation sites for CaP growth on AM PLA scaffolds. The plasma treatment increased amine functionalization, which promoted the chelation of Ca^2+^, thereby facilitating the deposition of CaP minerals onto the scaffold.

**Processing Temperature Considerations:** Processing temperature is a critical factor to consider in the surface engineering of polymeric materials, as they are heat-sensitive and prone to thermal degradation. Excessive temperatures can lead to the loss of physical, mechanical, and functional properties.^136^ Since degradation temperatures vary according to the polymer’s composition and molecular structure,^137^ coating methods should be carefully selected to match the thermal properties of the polymer.

Notably, degradation can occur even at temperatures as low as the polymer’s glass transition temperature (*T*_g_),^138–140^ which is the temperature at which the polymer transitions from a rigid, glassy state, to a rubbery, flexible state.^141^ Some conventional methods for coating CaP onto bone scaffolds, such as thermal spraying, sol–gel deposition, and plasma spraying often exceed the melting temperature (*T*_m_) of most thermoplastics. This includes both PLA (130–180 °C) and PCL (55–60 °C),^142,143^ which are among the most widely used polymers for AM biomedical scaffolds and devices, as indicated by the literature search described in the Supporting Information. This excessive heat may lead to deformation and structural instability, particularly for tissue engineering scaffolds that require precise porosities and geometries.

To reduce the effects of thermal degradation, it is ideal to maintain processing temperatures below the polymer’s *T*_g_ and *T*_m_. Accordingly, the methods reported in Table 1, such as SBF immersion and dip coating, have been performed near room temperature, making them suitable for polymeric scaffolds. Additionally, as stated by Liao et al.,^131^ cold spraying operates at lower temperatures than conventional thermal spraying, making it a viable alternative for polymers that are particularly temperature-sensitive.

##### 3.2.2.2 Organic Component

The organic matrix of bone comprises approximately 10% noncollagenous proteins and around 90% collagen, with COL I making up the majority (∼95%) of the collagen component.^144,145^ While the exact function of noncollagenous proteins is not yet fully understood, they have shown potential in supporting cellular behavior and contributing to the mechanical properties of bone.^146,147^

On the other hand, COL I, secreted by osteoblasts, provides critical structural support to bone tissues.^125^ It plays a vital role in tissue organization, influences the mechanical properties of bone, and serves as a scaffold for bone cells.^124,125^ Moreover, COL I directly regulates bone mineralization by controlling the deposition of inorganic minerals, such as HA, which enhances the bone’s strength and rigidity.^145,148^ In comparison to noncollagenous proteins, collagen-based materials have been more widely studied due to their abundance in the organic bone matrix.^147^

Notably, only one study by Teixeira et al.^27^ focused on using an ECM-mimicking organic component for surface engineering of AM polymeric scaffolds, specifically investigating the effects of COL I on the bioactivity of AM PLA scaffolds. This study, also referenced in Section 3.1.2, explored the pretreatment strategy in which PDA was used to facilitate the deposition of COL I onto PLA.^27^ Importantly, PDA functionalization also played a crucial role in enhancing the scaffold’s bioactivity by increasing surface hydrophilicity and providing additional bioactive functional groups that promoted cell adhesion.^27^ By improving the scaffold’s binding efficiency with COL I, PDA directly enhanced the biological effects arising from COL I, and in turn, significantly increased the cell viability of PDA/COL I coated scaffolds (Figure 8).

**Figure 8:**
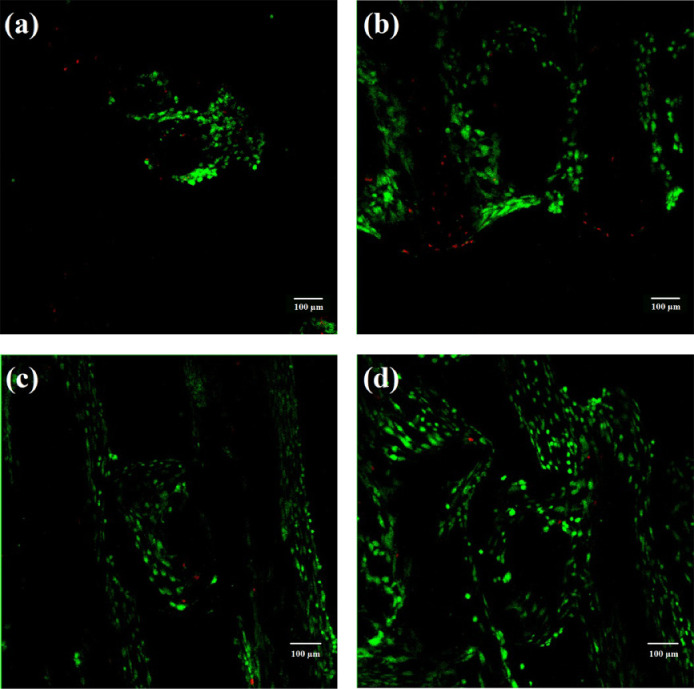
Cell viability after 7 days of culture on PLA scaffolds: (a) untreated; (b) COL I coated; (c) PDA coated; (d) PDA/COL I coated. Viable cells are shown in green, while nonviable cells are shown in red. Reproduced with permission.^27^ Copyright 2018, John Wiley and Sons.

The combination of PDA and COL I provided optimal conditions for early stage mesenchymal stem cell (MSC) response (within the first 7 days).^27^ MSCs, which can differentiate into bone-forming cells,^149^ also exhibited enhanced ECM deposition during the first 14 days. This suggests that the scaffolds supported MSC differentiation into osteoblasts, as ECM deposition is part of this process.^150^ By day 21, although the behavior of the MSCs appeared to be similar for both uncoated and coated scaffolds, cells seeded onto the coated scaffolds produced substantially higher amounts of alkaline phosphatase, confirming the coating’s ability to promote osteoinductivity.^27^ Accordingly, MSCs are more responsive in the long term to the ECM components that they themselves have secreted within the scaffold than to the proteins coated onto the biomaterial surface.^27^ This explains why the best conditions were obtained from the scaffold in the period of the early stage cell response

##### 3.2.2.3 Growth Factors

In addition to providing structural support, the ECM serves as a reservoir for a wide variety of bioactive molecules, including growth factors, cytokines, and other signaling molecules, all of which play pivotal roles in regulating stem cell growth and differentiation.^151^ The ECM delivers essential biochemical cues that coordinate various signaling pathways, thus mediating interactions between cells within tissues and organs that directly influence bone regeneration.^152,153^ Compared to biophysical cues, biochemical signals are generally more controllable and easier to deliver, making them highly effective for manipulating stem cell behavior.^153^

Among these signaling molecules, Bone Morphogenetic Proteins (BMPs), particularly BMP-2 and its recombinant form rhBMP-2, are notable growth factors that have received FDA approval for clinical use.^154,155^ BMPs are naturally occurring proteins found in the bone matrix,^156^ and have been shown to enhance the osteoinductivity of bone scaffolds and implants by directly stimulating osteogenesis.^157,158^

Nonetheless, their use has raised safety concerns due to numerous adverse effects reported, primarily linked to the rapid or excessive release of the protein and in dosages that exceed those naturally occurring in the body.^154,159^ To enhance the effectiveness of BMP-2 in bone regeneration, various strategies including molecular engineering, biomaterial modification, and synergistic therapy have been developed to optimize the sustained and controlled delivery of the protein.^160,161^ These approaches aim to ensure that BMP-2 is delivered in controlled therapeutic dosages over an extended period, thereby promoting healing while minimizing the risks associated with high doses and uncontrolled release.^159^

For AM polymeric scaffolds, controlled and sustained delivery of BMPs has been achieved through various surface coating strategies, including the use of PDA and PDA/HA coatings.^30,84^ In particular, PDA coating has enabled the sustained delivery of rhBMP-2 over 28 days with minimal burst release.^84^ Similarly, Park et al.^30^ showed that PDA/HA coated AM PCL scaffolds enabled the sustained delivery of BMP-2 for 21 days. One notable limitation in both studies is that cellular activities were primarily tested within a short time frame, such as up to 7 days, even though the BMPs were being released over a period of 21 to 28 days. While these studies have shown that increasing release of BMP concentration over long periods enhanced cellular responses, the exact threshold for optimal BMP dosages within a safe range have not been clearly defined. Thus, future studies should pinpoint the optimal BMP dosage for achieving sustained cellular responses, which is important as excessive amounts have been shown to induce adverse effects such as the formation of ectopic bone and hematoma.^31,162^

To further evaluate the effectiveness of BMP-2 in bone regeneration, Garot et al.^31^ conducted an *in vivo* study using BMP-2-loaded AM PLA scaffolds to repair critical-size bone defects in sheep. The scaffolds were coated with PEI/poly-l-lysine (PLL)/hyaluronic acid multilayer films, which were cross-linked using agents such as 1-ethyl-3-(3-(dimethylamino)­propyl) carbodiimide (EDC). This cross-linking stabilized BMP-2 and enabled its controlled, localized release.^31^ By adjusting the degree of cross-linking, the release profile of BMP-2 could be fine-tuned, enabling precise control over its delivery to the defect site.^31^ This approach significantly reduced the required BMP-2 concentration in the scaffold to around 120 μg/cm^3^, a 12-fold decrease compared to the 1500 μg/cm^3^ typically used with collagen sponges in clinical settings.^31^

In addition to the critical role of the multilayer film in BMP-2 delivery, Garot et al.^31^ also explored the impact of different scaffold pore geometries (Cubic, Gyroid, and Cubic–Gyroid) on the repair of critical-size bone defects in sheep. Each geometry was evaluated at two scales, denoted as S (small) and L (large), reflecting variations in pore dimensions. Preliminary findings showed that the Cubic S geometry resulted in the greatest amount of newly formed bone, indicating its potential as an optimal scaffold design for critical-size defect repair.^31^

However, the study did not isolate or assess the effects of specific architectural parameters such as porosity, pore size, interconnectivity, and pore shape. These factors were collectively grouped under the broad term “pore geometry”, which limited the ability to determine the individual contributions of each characteristic. Notably, the Cubic L design was excluded from further analysis due to its excessively large pore size, which rendered the scaffold too mechanically fragile for surgical handling.^31^ A more detailed analysis of the above-mentioned parameters would provide clearer insights into their individual contributions to the enhanced bone regeneration observed with Cubic S scaffolds.

After the preliminary results indicated the superior performance of Cubic S, Garot et al.^31^ introduced Gyroid S for comparison, as it possesses a similar pore size to Cubic S (Cubic S: 0.87 mm; Gyroid S: 0.8 mm). The main difference between the two geometries was in their pore shape, which allowed for a more effective comparison of the impact of pore shape on bone regeneration. Despite the similar pore sizes, Cubic S provided a more favorable environment for bone formation, resulting in more consistent and faster bone regeneration.^31^ Specifically, Cubic S scaffolds had a significantly higher surface-to-volume ratio (1.8 vs 1.35 for Gyroid S), offering more surface area available for cell adhesion and subsequent bone formation.^31^ This comparison proves the important role of pore shape in scaffold performance, even when pore sizes are comparable. Nonetheless, future research should address the individual effects of pore size, pore shape, interconnectivity, and porosity, as these factors contribute to tissue regeneration in distinct ways, and optimizing each of them is important for improving scaffold performance.^163–166^

In addition to full-length BMP-2, recent research has explored the potential of specific BMP-2-derived peptide segments, such as GBMP1α, to enhance scaffold bioactivity. Cassari et al.^32^ grafted this peptide onto PEEK using two different chemical approaches, i.e., amino-oxy groups via oxime formation and azido groups via photoactivation. Both strategies enabled stable peptide grafting, resulting in significantly improved osteogenic outcomes.

The incorporation of BMPs into AM polymer scaffolds offers a promising aid to enhance bone regeneration, as they are among the most potent osteoinductors available. However, to maximize their effectiveness while minimizing adverse effects, strategies such as controlled delivery through coatings and optimization of pore architecture should be carefully considered.

#### 3.2.3 Functional Materials

This section explores functional materials, which feature inherent functionalities to improve not only the biological properties but also specific characteristics such as mechanical performance, antibacterial activity, and modulation of the inflammatory response in AM polymeric devices. While these materials may not directly replicate the bone ECM, they provide significant advantages in improving the functionality of polymeric scaffolds. These functional materials are typically applied as coatings on AM polymeric scaffolds and devices, as outlined in Table 2.

**Table 2:** Functional Materials Utilized for Enhancing the Performance of AM Polymeric Bone Scaffolds and Devices

Functional material	Coating method	Main effect	Ref.
Mesoporous bioactive glass (MBG)	Dip coating	Enhances bioactivity	^33^
Cerium oxide (CeO_2_)	Multilayer coating (alkali hydrolysis, followed by PEI, CA and CeO_2_)	Enhances antibacterial properties; Modulates inflammatory response	^34^
Graphene oxide (GO)	Multilayer coating (PDA followed by GO)	Enhances bioactivity	^85^
Sericin	Plasma treatment followed by sericin	Enhances bioactivity	^25^
Calcium carbonate (CaCO_3_)	Pressure-assisted coating	Enhances mechanical performance; Modulates inflammatory response	^35^
Nickel	Electroless plating	Enhances mechanical performance	^167^
Aluminum	Radio frequency sputtering	Enhances mechanical performance	^168^

As shown in Table 2, coatings incorporating MBG, GO, and sericin, although extraneous to the human body, have demonstrated potential in improving the bioactivity of AM polymeric scaffolds. Specifically, MBG enhanced the surface roughness and hydrophilicity of AM PHBHHx scaffolds, promoting cellular activity through the release of Ca^2+^ and silicon ions in cell culture media.^33^ Similarly, GO boosts bioactivity by increasing surface roughness and hydrophilicity, while its oxygenated groups further enhance interactions with biomolecules, supporting osteogenesis.^85^ Sericin, a silk protein, plays a key role in promoting HA nucleation on the scaffold’s surface.^25^ The side chains of aspartic acid and glutamic acid in sericin provide sites for electrostatic attraction with Ca^2+^, followed by the formation of bonds with PO_4_^3–^, initiating HA deposition.^25^ Moreover, sericin also promotes surface hydrophilicity, leading to improvement in cell adhesion, proliferation, and osteogenic differentiation, making it a promising material for bone tissue engineering applications.^25^

In addition to bioactivity enhancement, functional coatings have also been used to impart specific properties including mechanical strength, antibacterial properties, and inflammatory response modulation in AM polymeric scaffolds, as discussed in the following sections.

##### 3.2.3.1 Mechanical Performance

Synthetic polymers are often recognized for their superior mechanical properties and ease of processing through AM as compared to natural polymers, leading to their wider adoption for the AM of bone scaffolds.^169,170^ However, it is important to emphasize that commonly used synthetic polymers, such as PLA and PCL, are generally better suited for creating porous, degradable scaffolds intended for bone regeneration, rather than for permanent, load-bearing implants.^42^ For example, AM scaffolds made from PLA, PCL, and PBAT have shown promise in supporting the regeneration of cancellous bone.^27,34,129^ As shown in Figure 9, the cancellous bone found in the inner part of bones, has a spongy, highly porous structure. In contrast, the cortical bone which forms the dense outer layer is much stronger and less porous.^171^

**Figure 9:**
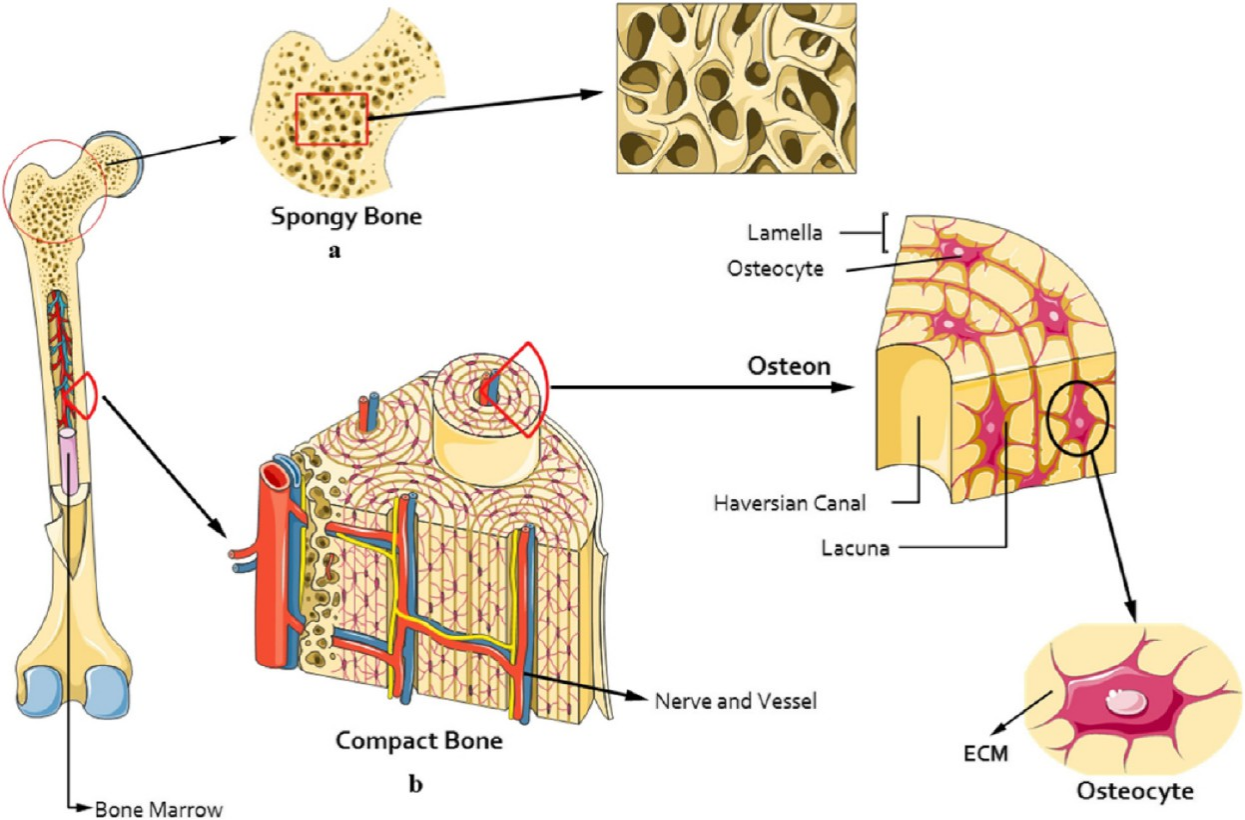
Illustration of bone structure showing compact (cortical) and spongy (cancellous) bone. Reproduced from ref 172. Available under a CC-BY license. Copyright 2022.

As shown in Table 3, pure PLA scaffolds, even without surface modifications, can achieve mechanical properties comparable to those of cancellous bone when their porosity is carefully tailored.^27,34^ The incorporation of bioceramics, such as HA/bioglass,^129^ (cited in Section 3.2.2 as part of surface modification incorporating inorganic components mimicking ECM) and CaCO_3_, further enhanced the mechanical properties of PBAT and PLA, respectively, bringing them closer to the properties of cancellous bone.^35,129^ By adjusting the porosity and applying appropriate coatings, synthetic polymer-based scaffolds can closely mimic the mechanical properties of cancellous bone, thus demonstrating strong potential for bone regeneration applications.

**Table 3:** Comparison of Properties between Cancellous Bone and Polymeric Scaffolds for Potential Cancellous Bone Applications

Material	Porosity (%)	Stiffness (GPa)	Compressive strength (MPa)	Compressive modulus (GPa)	Ref.
Cancellous bone	50–90	0.01–0.5	0.1–15	0.12–1.1	^47^,^173^
PLA (uncoated)	∼60	∼0.4	∼9.5	n/a	^27^
PLA (uncoated)	∼68	n/a	n/a	0.03	^34^
HA/bioglass-coated PBAT	∼57	∼0.04	∼2.8	n/a	^129^
CaCO3 coated PLA	n/a	n/a	n/a	0.15	^35^

Nonetheless, while degradable scaffolds may be beneficial for younger patients with high tissue regeneration rates, they may be less suitable for older patients, whose slower tissue regeneration may prevent the restoration of functionality in the damaged area.^173^ In such cases, patients may require permanent bone implants or substitutes to provide long-term structural support.^173^ Currently, metallic implants remain the gold standard for providing long-term, load-bearing functionality in defects or fractures at load-bearing sites, such as the femur and tibia, due to their superior mechanical strength.^173,174^ However, their significantly higher stiffness compared to bone can lead to stress shielding, a phenomenon where a highly stiff implant prevents normal bone loading, which in turn reduces the bone density.^175^

In response to this, PEEK is a high-performance polymer that has emerged as a promising alternative to metal implants. PEEK offers high strength and stiffness that are closer to, but still somewhat lower than, those of human bone.^41^ However, its properties can be further enhanced through surface treatments or by combining it with other biomaterials to make it more comparable to human bones.^41,176,177^ As a result, PEEK has great potential in reducing the risk of stress shielding while maintaining mechanical properties suitable for load-bearing applications.^178,179^ However, the high cost and processing challenges associated with PEEK—due to its high melting temperature (350–400 °C) and melt viscosity—require specialized printers for fabrication.^180,181^ This makes more processable polymers, such as PLA, still of significant interest for exploration.

One promising solution to bridge the gap between the mechanical properties of metals and those of synthetic polymers is the use of metallic surface coatings on polymer substrates. For instance, nickel deposition on PLA through electroless deposition and electroplating has been shown to improve the compressive strength of PLA components.^167^ While the increase in strength is not yet substantial enough for load-bearing applications, this approach has potential for improving PLA’s mechanical properties while maintaining its low density, which is beneficial for medical implants where weight is a concern.

Another issue faced by polymers in terms of implant longevity is their tendency to degrade in the body over time. While this is a key advantage when natural tissues are expected to replace the implant, biodegradation directly deteriorates the mechanical properties of polymer-based components.^182^ It has been shown that metallizing PLA with an aluminum layer, deposited through radio frequency sputtering, forms an oxide barrier on the surface of PLA. According to Aktitiz et al.,^168^ this oxide layer can potentially increase the polymer’s resistance to degradation, helping preserve its mechanical properties for a longer period. Although metallization of AM polymeric biomedical devices is still a relatively new approach and the specific metal involved requires careful selection, it has great potential for enhancing both the mechanical properties and degradation resistance of polymeric implants.^183^

It should be emphasized that the concern regarding inadequate mechanical properties of synthetic polymers typically arises in load-bearing applications. It is envisaged that enhancing the mechanical properties of synthetic polymers can potentially extend their applicability to more demanding structural applications, beyond scaffolds for tissue engineering.

##### 3.2.3.2 Antibacterial Properties

Implant-associated infection remains a significant concern in bone implant procedures. A study published in 2024 on total hip and knee arthroplasty found infection to be one of the leading causes of revision surgery.^184^ While this data applies to permanent implants, tissue scaffolds and biomaterials also face infection risks due to the compromised host defense caused by foreign materials.^185^

Kuijer et al.^186^ were among the first to explore the potential difference in infection susceptibility between traditional implants and tissue engineering scaffolds. Based on the “race for surface” concept which suggests that tissue cells and bacteria compete for attachment to the surface of implants,^187^ they hypothesized that tissue scaffolds that are precolonized with mammalian cells may be less susceptible to infection, as the presence of mammalian cells may limit bacterial colonization. On the other hand, the porous structure of tissue scaffolds may increase the surface area for bacterial attachment, potentially making them more prone to infection.^186^ Nonetheless, their results demonstrated that traditional implants and tissue scaffolds, whether or not seeded with cells, have similar susceptibility to infection.^186^ This highlights the need for tissue scaffolds—especially polymeric scaffolds without inherent antibacterial properties,^118,188^ to also be antibacterial in addition to their primary tissue regeneration function. For clarity and conciseness, the term “implant” in this section encompasses both implants and tissue scaffolds, as both are implanted in the body and have similar infection risks.

Bone infections are primarily caused by *S. aureus*, a Gram-positive bacterium responsible for up to two-thirds of bone implant infections.^189^ The mechanisms by which *S. aureus* causes bone infections, including its ability to adhere to implants and form biofilms, have been extensively explored by Masters et al.^189^ In brief, the first critical step in implant-associated infections is bacterial adhesion. If bacteria successfully adhere to the implant before tissue regeneration occurs, the host’s defense mechanisms are often unable to prevent the subsequent biofilm formation.^190^ Biofilm formation is a complex, multistep process.^191^ Once bacteria have adhered, they proliferate, aggregate, and form microcolonies.^191^ These microcolonies then mature into an intricate, 3D structure, acquiring a “mushroom” or “tower” shape.^191^ Upon maturation, biofilms can rupture, releasing bacteria that initiate a new cycle of biofilm formation. Once established, biofilms lead to chronic infections, as they act as reservoirs for bacterial communities that continuously shed bacteria in the body, prolonging infection.^192^ Moreover, biofilms are notoriously difficult to treat with antibiotics due to their complex structure, which impedes the penetration of therapeutic agents.^193^ As a result, implant-associated infections often require surgical removal of the infected bone and implant.^194^ This can lead to complications and prolonged healing times, as revision surgeries often involve difficult interventions.^194^

As mentioned earlier, bacterial attachment to implant surfaces is a critical step in the development of infection. Surface properties such as topography, charge, and wettability can be tailored to enhance the antibacterial characteristics of implants, making them less prone to bacterial adhesion.^195^ However, relatively few studies have focused on improving the antibacterial properties of AM polymeric devices through surface engineering. For instance, one study (cited in Section 3.2.1 as part of the physical surface modification strategies) explored the use of femtosecond laser treatment to introduce microchannels on the surface of AM PCL scaffolds, which successfully disrupted *S. aureus* adhesion.^112^ Another study demonstrated that decorating AM PLA scaffolds with CeO_2_ nanoparticles exhibited antibacterial effects against *S. aureus*, likely due to electrostatic interactions between the nanoparticles and the negatively charged bacterial cells.^34^ Additionally, the widely used pretreatment strategy of PDA coating has shown antibacterial activity against *S. aureus*,^26^ as previously discussed in the pretreatment strategy section. It is worth noting that PDA’s antibacterial properties are well-established and widely attributed to its ability to act as a barrier, blocking bacterial nutrient supply, while its active catecholic groups disrupt the bacterial cell membrane, altering its permeability and ultimately causing bacterial cell death.^196^

While significant attention has been given to enhancing the tissue regenerative properties of AM polymeric scaffolds, the antibacterial aspect remains relatively underexplored. Given the critical role of infections in hindering the healing process, the antibacterial properties of these implants should be assessed alongside their bioactivity to ensure successful healing outcomes.

##### 3.2.3.3 Inflammatory Response Modulation

Bone regeneration occurs in three main stages: (i) inflammatory, (ii) reparative, and (iii) remodeling stages. After an injury, blood from ruptured vessels coagulates into a mass (hematoma).^197^ Inflammation begins immediately, recruiting the necessary cells to clear debris and stimulate bone healing at the injury site.^198,199^ In the reparative phase, the hematoma is replaced by a soft callus composed of connective tissue, blood vessels, cartilage, and spongy bone.^197^ Osteoblasts then invade the soft callus, depositing new tissue and mineralizing it into a hard callus of woven, immature bone.^197^ Finally, in the remodeling phase, the bone reshapes itself into a mature, mechanically stable structure to meet functional demands.^197^

Similarly, the implantation of foreign materials, whether bioinert or nontoxic, triggers a series of immune and repair-related responses that are collectively known as the foreign body response (FBR).^200,201^ As thoroughly described in the literature,^200,202^ this process unfolds in several stages that can be summarized as follows. The initial stage involves the adsorption of proteins from blood and interstitial fluids to the surface of the implant. Following this, neutrophils are recruited to the site, followed by macrophages that work to eliminate potential threats and mediate tissue repair. At this point, the inflammatory response is similar to that of a typical injury. However, as macrophages accumulate at the implantation site, the acute inflammatory response develops into the FBR. Macrophages attempt to phagocytize and degrade the implant. If the implant is successfully degraded, the FBR resolves, and tissue slowly returns to normal. If the implant is too large and degradation is prolonged, the acute response progresses into chronic FBR. During this phase, macrophages release cytokines that induce the formation of a fibrous capsule around the implant, which acts as a physical barrier, thereby hindering the implant’s interaction with surrounding tissues and consequently impairing its functionality. Furthermore, macrophage membranes may fuse to form foreign body giant cells (FBGCs) at the implant surface in an attempt to engulf the larger material. The presence of FBGCs is a hallmark of chronic inflammation which hinders the healing process.^200,202^

In the case of bone tissue engineering, inflammation is essential for initiating healing, but excessive or prolonged inflammation can significantly compromise this process.^203^ To address this, surface coatings have been explored to modulate immune and inflammatory responses in AM polymeric implants. For instance, coating the surface of AM PLA scaffolds with CeO_2_ nanoparticles has shown promising results in reducing the oxidative stress caused by reactive oxygen species (ROS), which can exceed the optimal levels required for tissue regeneration due to inflammation at bone defect sites.^34^ CeO_2_ nanoparticles are particularly effective because they possess a large number of oxygen vacancies, allowing them to alternate between cerium III (Ce^3+^) and cerium IV (Ce^4+^) oxidation states.^34^ This enables them to scavenge ROS, thereby creating a favorable environment for osteogenesis.

Additionally, hydrogel coatings based on six-arm star-shaped NCO-poly­(ethylene oxide-*stat*-propylene oxide) (sP­(EO-*stat*-PO)) have been shown to permanently functionalize PCL scaffolds with hydrophilic properties.^204^ This modification minimizes nonspecific protein adsorption, which is crucial because this can attract and activate macrophages, potentially triggering inflammatory responses at implantation sites.^204^

Another concern is the acidic degradation products released from polymers like PLA, which can induce inflammation by causing abrupt pH shifts in the surrounding tissues. The problem can be exacerbated by thermally induced degradation caused by high-temperature exposure in common AM techniques like FFF.^205,206^ To mitigate this, research by Donate et al.^35^ demonstrated the potential of CaCO_3_ coatings to buffer the acidic byproducts of AM PLA degradation. These coatings release CaCO_3_ particles that counteract the pH decrease caused by PLA’s acidic byproducts, potentially minimizing inflammatory responses.^35^

While the above-mentioned surface modifications have shown promise in modulating inflammatory responses, they do not directly relate to FBR. Hence, this area remains underexplored in AM polymeric scaffolds. Furthermore, while bioactive coatings can promote bone healing, their interaction with allogeneic stem cells requires careful consideration due to potential immune responses.^81^ Therefore, continued research in these areas is essential, particularly to better understand how design factors (e.g., shape, stiffness, porosity) and surface properties (e.g., roughness, hydrophilicity, charge) of AM polymeric implants can effectively modulate FBR and enhance tissue regeneration while minimizing undesirable immune reactions.^200,207^

### 3.3 Other Tissue Engineering Applications

While the largest portion of the reviewed papers is devoted to bone tissue engineering, other fields of tissue engineering have also been explored in the literature, including vascular, skin, cartilage, and skeletal muscle tissue engineering. Due to the relatively limited number of articles available in these areas, it was not possible to subcategorize the surface modification techniques as was done for bone-related applications. Instead, this section highlights the key objectives and achievements of research in surface engineering for these less-explored areas.

#### 3.3.1 Vascular Tissue Engineering

In the reviewed literature, two studies, both published in 2019, applied surface engineering to enhance the usability of PCL scaffolds in vascular tissue engineering.^107,208^

In the first study, Lee et al.^208^ developed an artificial vascular scaffold addressing issues related to inadequate mechanical strength and limited bioactivity of vascular grafts. The scaffold featured a bilayer design (Figure 10), wherein a PCL nanofibrous tubular structure resembling native vascular tissues was fabricated by electrospinning, followed by the deposition of AM PCL polymer strands. Along with the tubular structure, the incorporation of AM polymer strands was crucial to achieving mechanical properties comparable to human blood vessels.^208^ Strictly speaking, while the contribution by Lee et al.^208^ does not describe the surface modification of an AM polymer device, this hybrid approach demonstrates the potential of combining AM with other fabrication techniques to create mechanically robust vascular grafts and, for this reason, it was included in this review.

**Figure 10:**
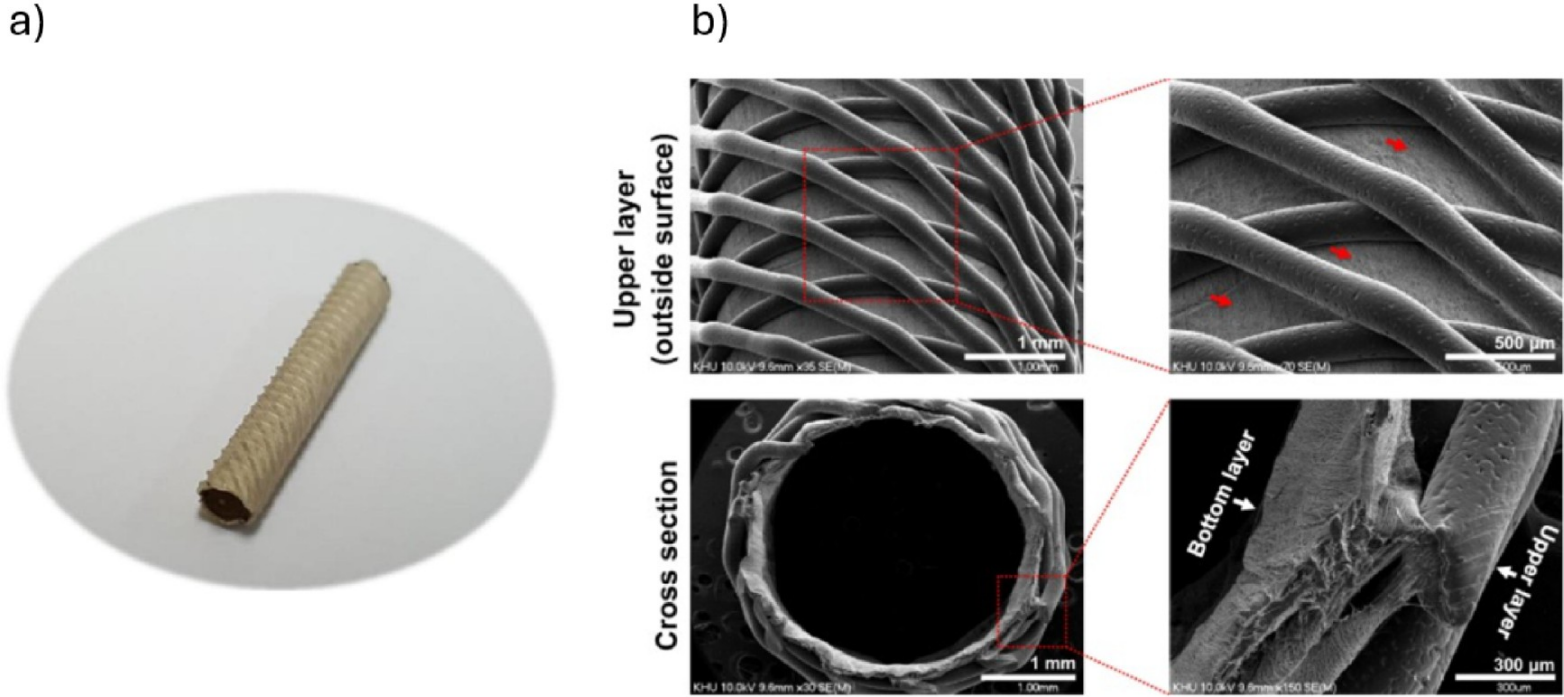
(a) Artificial vascular scaffold developed by Lee et al.,^208^ and (b) its scanning electron microscopy images. Red arrows indicate the highly aligned electrospun nanofibers. Reproduced with permission from ref 208. Copyright 2018 Elsevier.

The approach proposed by Lee et al.^208^ is also relevant because, in order to optimize the scaffold’s bioactivity, the electrospun scaffolds were pretreated with PDA, followed by immobilization with vascular endothelial growth factor (VEGF). In this case, PDA effectively bound the VEGF polypeptide to the scaffold, while also increasing hydrophilicity.^208^ Notably, VEGF plays a crucial role in nearly every phase of vascular tissue development and maintenance.^209^ It has been shown to enhance endothelial cell migration, proliferation, and differentiation, contributing to both vasculogenesis and angiogenesis.^209^ As expected, the combination of the hydrophilicity induced by PDA and VEFG-mediated biological stimulation promoted the *in vitro* proliferation of both smooth muscle and endothelial cells, and favored angiogenesis of the scaffold.^208^ Collectively, the optimal mechanical properties combined with the biological enhancement of the bilayered scaffold make it a promising candidate for vascular tissue engineering.

In a second study, Kim et al.^107^ incorporated small molecular therapeutic agents into a vascular scaffold specifically designed to treat carotid artery stenosis, a condition caused by the narrowing or blockage of the carotid artery. This disease is commonly linked to excessive levels of low-density lipoprotein (LDL) cholesterol (“bad cholesterol”), leading to plaque buildup within the arteries.^210^ The treatment for this condition typically involves antiplatelet medications such as aspirin and clopidogrel, along with cholesterol-lowering drugs like statins, including atorvastatin and simvastatin.^211^ For this reason, Kim et al.^107^ developed a drug-coated PCL scaffold, intended to support a localized delivery of drugs for preventing blood clots and also lowering the LDL cholesterol levels in patients. The scaffold was coated with a combination of aspirin and atorvastatin calcium salt, with oxygen plasma pretreatment playing a critical role in enhancing the adherence of the drug coating by increasing surface hydrophilicity.^107^ The study demonstrated successful drug coating on PCL scaffolds, laying an important foundation for future research in this area.

#### 3.3.2 Adipose Tissue Engineering

In the reviewed literature, only one study focused on adipose tissue engineering, i.e., by Jain et al.^212^ published in 2020. The main objective of this study was to design a scaffold with appropriate mechanical properties and bioactivity to support soft tissue engineering, particularly adipose tissue. In this context, polymers like PLA and PCL are rarely used due to their inherent stiffness and brittleness.^212^ However, Jain et al.^212^ still sought to leverage the mechanical strength and processability of these polymers. To overcome the limitations of PLLA, a stereoisomer of PLA,^206^ these researchers utilized poly­(l-lactide-*co*-trimethylene carbonate) (PLATMC), a copolymer that combines l-lactide with trimethylene carbonate (TMC) that imparts flexibility, making PLATMC suitable for soft tissue engineering applications.^212^

To impart adequate mechanical properties to PLATMC scaffolds, Jain et al.^212^ emphasized the importance of optimizing the 3D bioprinting parameters such as print pressure and print speed, which directly influence polymer degradation during the printing process. Polymer thermal degradation is an inherent part of printing, however, Jain et al.^212^ noted that a certain level of degradation (i.e., a 57% decrease in the molecular number (Mn) of PLATMC) was necessary to achieve a printable viscosity. Excessive degradation, however, compromised the mechanical integrity of the scaffold.

In addition, the study also investigated the effects of scaffold geometry, comparing direct pores (i.e., pores aligned vertically throughout the scaffold layers) and strand shift pores (i.e., pores offset between layers).^212^ Direct pores demonstrated superior mechanical properties due to better stress distribution, while strand shift pores created an environment more conducive to cell adhesion by increasing surface area and pore interconnectivity.^212^ Finally, the bioactivity of the scaffolds was enhanced through PDA coating, which improved hydrophilicity. This improvement facilitated the attachment, proliferation, and differentiation of human adipose tissue-derived stem cells, making the scaffolds a promising candidate for adipose tissue engineering applications.^212^

#### 3.3.3 Cartilage Tissue Engineering

Cartilage is found in various parts of the body, including joints such as the elbows, knees, and ankles.^213^ While it is firm, it is much softer and more flexible than bone.^213^ Two studies published between 2021 and 2022 focused on cartilage tissue engineering.^36,214^ Notably, both contributions highlighted the suitability of polyurethane (PU),^36,214^ and its thermoplastic variant, TPU,^36^ for this application. Both PU and TPU offer a combination of mechanical strength and flexibility that aligns well with the properties of cartilage, making them more suitable as cartilage scaffolds compared to brittle and rigid polymers like PLA.^36,214^

In the first study, PU scaffolds were specifically designed to assist the regeneration of the meniscus,^214^ a c-shaped cartilage pad in the knee that acts as a shock absorber.^215^ Deng et al.^214^ demonstrated that PU scaffolds with 25% of porosity possessed mechanical properties compatible with the human meniscus. As shown in Figure 11, the scaffolds demonstrated excellent *in vivo* biocompatibility, with no obvious signs of inflammation, redness, or swelling following implantation. Furthermore, histological analysis revealed tissue ingrowth and the formation small blood vessels within the scaffold, indicating good integration with the host tissue.^214^

**Figure 11:**
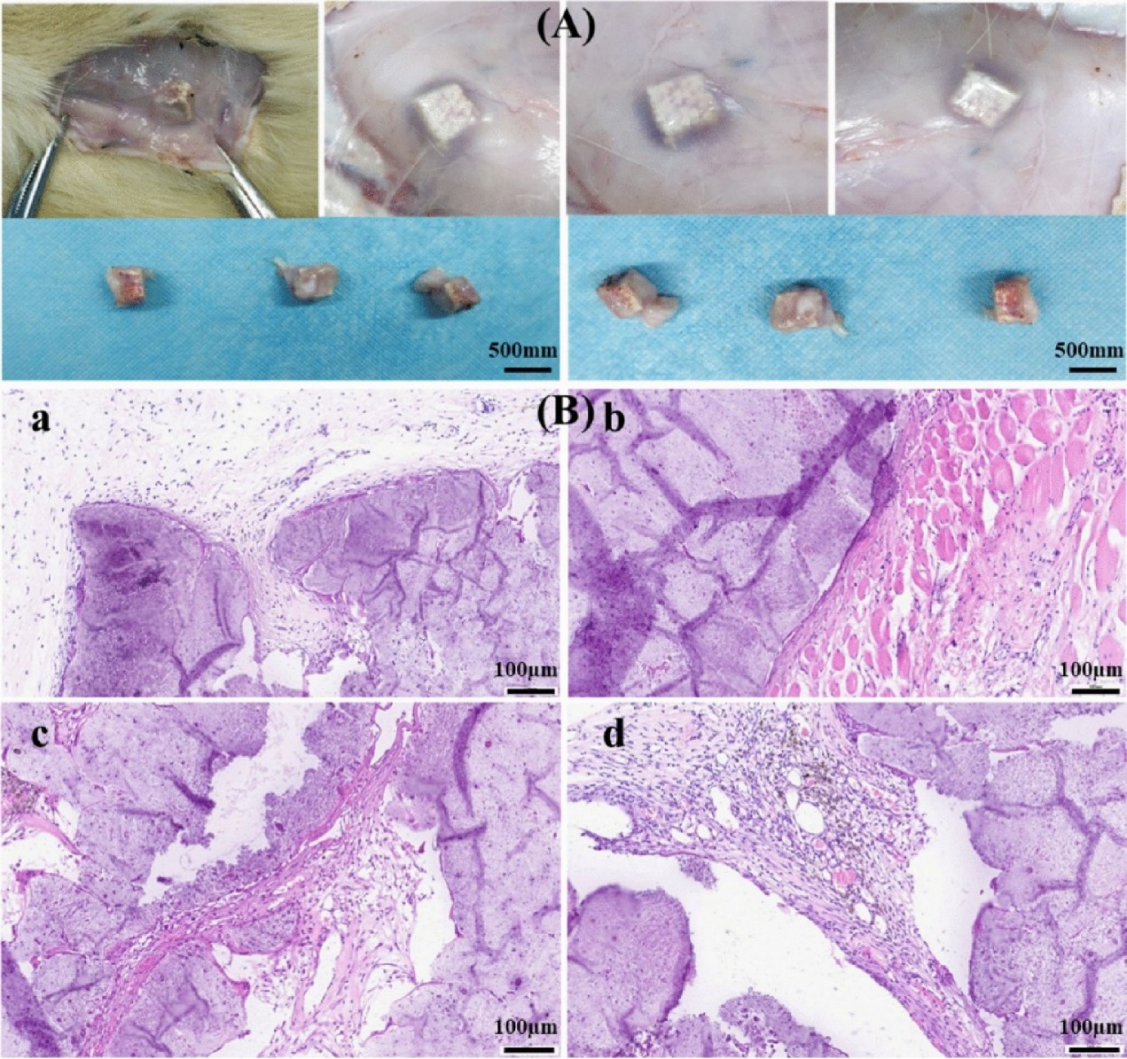
*In vivo* biocompatibility of PU scaffolds: A) macroscopic view showing no signs of inflammation around the subcutaneous tissues of the implanted scaffolds. B) Histological staining images of the tissues surrounding the implants (a and c), and interior tissues of the explants (b and d). Reproduced from ref 214. Available under a CC-BY license. Copyright 2021.

To facilitate cell adhesion and proliferation, Deng et al.^214^ further coated the scaffolds with either fibronectin (FN) or COL I. Between the two, FN promoted superior cell attachment, proliferation, and human MSC (hMSC) chondrogenesis. This effect was largely attributed to potentiated initial cell adhesion, driven by the interaction between α5β1 integrin—a cell surface receptor found on hMSCs—and the FN coating on the scaffold.^214^ These findings suggest that FN-coated PU scaffolds could play a key role in treating damaged menisci, as they combine the necessary mechanical and biological properties to support meniscus regeneration.

In the second study, Zhang et al.^36^ designed a TPU scaffold with mechanical properties comparable to natural cartilage constructs. They introduced a novel strategy to address the surface limitations of 3D printing, particularly the challenges in achieving high resolution microscale structures smaller than 100 μm and in maintaining adequate porosity without undermining the mechanical integrity of the scaffold. The new approach involved combining FFF with supercritical carbon dioxide (CO_2_) microcellular foaming.^36^ The foaming process began by saturating TPU filaments in supercritical CO_2_ within a high-pressure vessel. This process allowed CO_2_ to dissolve homogeneously within the polymer matrix. Subsequently, as shown in Figure 12, the heating of the CO_2_-saturated filaments during printing caused the absorbed CO_2_ to form bubble nuclei.^36^ Upon extrusion, the high temperature and the sudden drop in extrusion pressure prompted the expansion of these bubble nuclei. As a result, the bubble walls stretched and coalesced, creating interconnected structures with microcellular porosity. Through this method, Zhang et al.^36^ successfully fabricated hierarchical scaffolds, where macropores were created by FFF printing at the layer level, while micropores were introduced by microcellular foaming throughout the material. This combination led to a hierarchical structure consisting of interconnected pores of varying sizes.^36^

**Figure 12:**
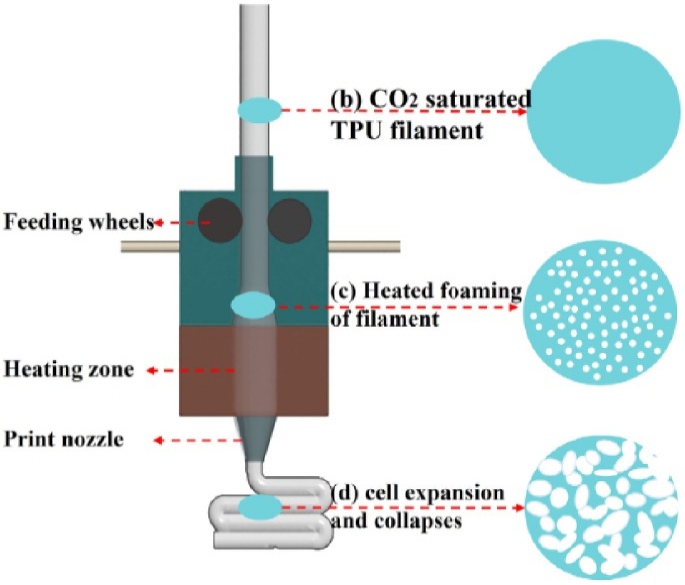
FFF printing of CO_2_-saturated TPU filaments. Adapted with permission from ref 36. Copyright 2022 John Wiley and Sons.

Remarkably, the microporosity introduced by the foaming process did not compromise the mechanical properties of the scaffold.^36^ Zhang et al.^36^ attributed the retained structural stability to the relatively small size of pores observed in FFF-printed parts using foamed filaments. In FFF printing, inter-raster voids (which can be considered as pores) typically form due to incomplete bonding between the deposited rasters (Figure 13).^216^ In contrast, with foamed filaments, the expansion of bubble nuclei during extrusion causes the rasters to expand upon deposition. This expansion reduces the size of inter-raster voids, offsetting the overall increase in pore density, thereby preserving the scaffold’s mechanical strength.

**Figure 13:**
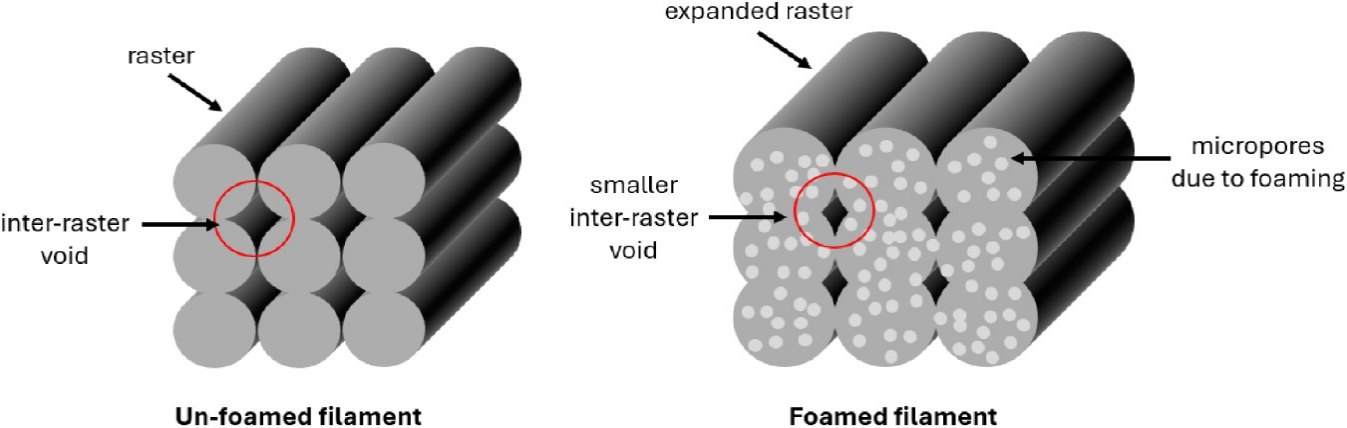
Illustration of FFF parts printed with unfoamed and foamed filaments.

From the biological perspective, the foaming process was shown to enhance cell attachment, as the presence of micropores increased the surface area available for cell adhesion.^36^ Meanwhile, the interconnected internal channels caused by FFF printing facilitated cell proliferation and differentiation within the scaffolds, enabling effective tissue regeneration.^36^ Moreover, the foamed structures allowed for better adherence of an antibacterial agent, which in this study was GO flakes with PDA acting as a robust bridging layer.^36^ Accordingly, the sharp morphology of the GO flakes effectively killed bacteria through mechanical contact, enhancing the scaffold’s antibacterial properties.^36^

#### 3.3.4 Skin Tissue Engineering

Alginate is a natural polymer widely used in wound healing and tissue regeneration due to its ability to closely mimic the physical structure of the ECM.^217^ However, alginate scaffolds face significant limitations, including rapid degradation and limited cell adhesion sites, which hinder effective cellular activities. In 2021, Khoshnood et al.^218^ addressed these challenges by coating 3D bioprinted alginate scaffolds with PEI. According to the study, PEI formed a strong polyelectrolyte multilayer on the negatively charged alginate substrate through electrostatic adsorption.^218^ This coating effectively delayed the degradation of alginate by creating a barrier layer, driven by the strong interactions between the amino groups in PEI and the carboxylate groups in alginate.^218^ Additionally, PEI enhanced the scaffold’s surface properties by significantly increasing hydrophilicity due to its abundant amino groups.^218^ The cationic nature of PEI also acted as an anchor for cells, further facilitating cell adhesion.^218^ Overall, these modifications improved the biocompatibility of the scaffolds and promoted better cellular interactions, addressing key shortcomings of using alginate for wound healing and tissue engineering applications.

#### 3.3.5 Skeletal Muscle Tissue Engineering

An important feature of scaffolds for effective skeletal muscle regeneration is their ability to promote the formation of highly aligned and densely packed myofibers. In this context, scaffolds characterized by anisotropic directional micropatterns or microgrooves have demonstrated great potential in promoting the alignment of myofibers, by guiding cell fusion and driving the formation of long and thick myotubes.^219–221^ This alignment is crucial for effective force transmission and contractility, enabling the regeneration of functional muscle fibers.^219^

In 2019, Miao et al.^222^ integrated FFF printing with a coating technique to fabricate thin-walled structures to support skeletal muscle tissue regeneration (Figure 14). A key feature of their approach is leveraging the staircase effect inherent to FFF printing to form “microchannels” within the scaffold, which provide anisotropic topographical cues that guide cell alignment, elongation, proliferation, and differentiation into myotubes. However, it is important to clarify that these microchannels are not directly printed using FFF. Rather, they emerge because of the coating process and the dissolution of the printed sacrificial template. Specifically, a sacrificial template was first printed using a poly­(vinyl alcohol) (PVA) filament, followed by the application of a PCL coating.^222^ Since the coating conforms to the geometry of the PVA sacrificial template, dissolving the template reveals the PCL based thin-walled structure with an embedded network of microchannels.

**Figure 14:**
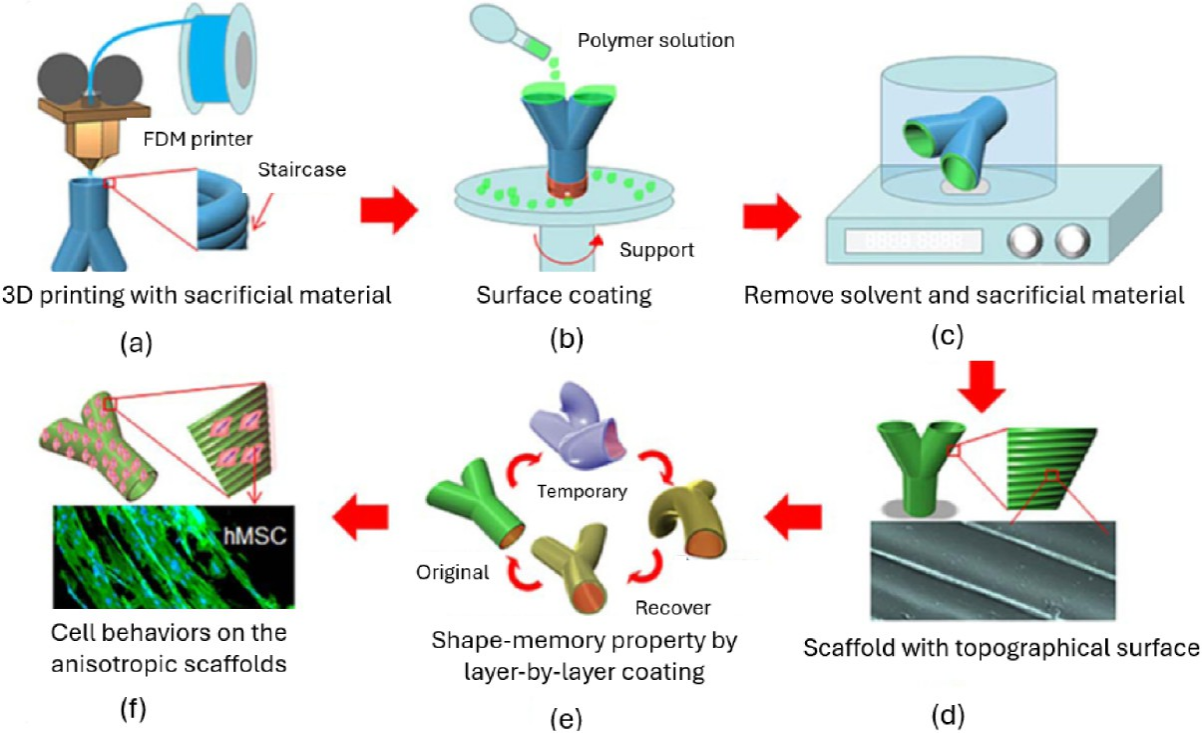
Workflow of Miao et al.^222^ to fabricate scaffolds with embedded microchannels. Adapted with permission from ref 222. Copyright 2019 IOP Publishing.

Notably, the sacrificial template was printed as a hollow structure with a 0% infill density.^222^ To prevent the breakage of the PCL thin-walled structure upon manipulation, the PCL solution was injected into the hollow section of the printed template rather than applied externally through immersion (Figure 14).^222^ This is because the PVA sacrificial template swells during dissolution, and this inevitably leads to the bursting of the PCL thin-walled structure when applied externally.^222^ Thus, by injecting the coating internally, Miao et al.^222^ successfully maintained the structural stability of the PCL thin-walled structure upon the dissolution of the sacrificial template.

Importantly, thin-walled structures offer the advantage of creating flexible scaffolds or implant to accommodate the dynamic biological environments of soft tissues like skeletal muscles, as these tissues require structures with high deformability to support their regeneration.^223^ However, FFF printing alone, even with flexible polymers, remains constrained by its minimum printable wall thickness, typically exceeding 200 μm due to the limitation of raster width associated with commercial FFF printers.^223^ Thus, the combination of FFF printing and a coating strategy offers a promising solution in fabricating highly flexible and structured scaffolds tailored for soft tissue engineering.

In terms of biological performance, Miao et al.^222^ demonstrated that optimizing the polymer coating concentration and adjusting the FFF print layer height allowed the PCL thin-walled structure to provide anisotropic topographical cues, effectively promoting myogenic differentiation and demonstrating strong potential for skeletal muscle regeneration. Furthermore, the polymer coating can be tailored to introduce shape memory effects, enabling the fabrication of 4D structures with programmable deformation.^222^ This property is particularly advantageous for minimally invasive surgical applications, as the scaffold can be designed to compactly fit into a target site and later expand to its original shape over time.^222^

Miao et al.^222^ further highlighted that this coating strategy addresses the limited availability of smart filaments for direct FFF printing. By modifying the polymer coating formulation and applying it onto FFF-printed sacrificial templates, it is possible to create structures with advanced functionalities. Nonetheless, in our opinion, developing smart filaments specifically for FFF printing presents distinct advantages, particularly in enabling efficient and scalable production of smart structures.^205^ The maturation of smart filament technology could streamline fabrication processes by eliminating multiple postprocessing steps required in the approach of Miao et al.^222^ However, FFF alone remains incapable of producing ultrathin-walled structures. Ultimately, the study by Miao et al.^222^ provides an innovative framework for enhancing the capabilities of FFF-printed scaffolds, expanding their potential applications in soft tissue engineering applications.

### 3.4 Surface Engineering for Applications Beyond Tissue Engineering

Surface engineering holds an important role in a wide range of biomedical applications beyond the scope of tissue engineering. Unlike therapeutic-focused applications, these surface modifications primarily aim to impart specific functionalities to AM devices in order to extend their applicability in areas such as diagnostics and drug delivery. This section explores the versatility of surface engineering and its ability to significantly expand the biomedical applications of AM polymeric devices beyond tissue engineering.

#### 3.4.1 Microfluidics

As mentioned in Section 2, LOC devices are key microfluidic systems that are driving advancements in the field of *in vitro* diagnostics, owing to their ability to integrate multiple laboratory functions onto a single chip (Figure 15).^224^

**Figure 15:**
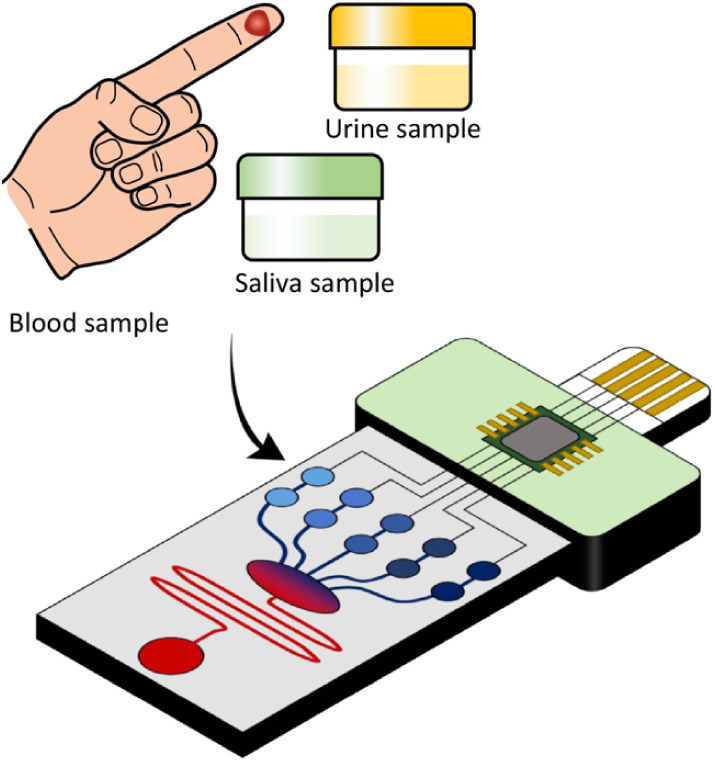
Illustration of LOC device for point-of-care applications. Adapted with permission from ref 225. Copyright 2015 Elsevier.

One crucial feature of LOC diagnostic devices is their ability to immobilize biomolecules, enabling the capture, detection, and interaction with specific biomolecules for accurate diagnostics.^226^ In this context, Pokharna et al.^17,18^ highlighted the importance of enhancing the hydrophilicity of AM PLA and acrylonitrile butadiene styrene (ABS) LOC devices to improve protein attachment. They evaluated three methods: hydrolysis, ultraviolet (UV) radiation, and gold thin film deposition.^17,18^ Among them, hydrolysis stood out as the most effective treatment. It significantly increased the surface hydrophilicity and generated abundant carboxyl groups on the surface, which readily reacted with the amino groups of the protein utilized.^18^ On the other hand, UV-treated surfaces showed limited protein attachment due to poor activation of carboxyl groups.^18^ Although the third method involving gold film deposition also led to desirable protein attachment, it was a more complicated process requiring a combination of both physical and chemical procedures.^18^ Thus, hydrolysis was finally identified as the most efficient method to improve the hydrophilicity of AM PLA and ABS surfaces for LOC applications.^18^ The mechanism through which hydrolysis enhances surface wettability has already been elaborated in the previous section (Section 3.1.1).

Brandhoff et al.^19^ also highlighted the importance of improving the hydrophilicity of resins for stereolithography (SLA) to produce surfaces that can repel nonspecific protein adhesion. This is particularly crucial during enzyme-linked immunosorbent assay (ELISA) applications, where nonspecific protein binding can interfere with the accuracy and reproducibility of the test.^227^ By making the surface hydrophilic due to a poly ethylene-glycol (PEG) layer, Brandhoff et al.^19^ also addressed other technical challenges like bubble formation, which can disrupt fluid flow in the microfluidic channels.

While hydrophobic coatings have not been extensively explored in the literature reviewed so far, their significance in microfluidic applications should not be overlooked. Hydrophobic surfaces are crucial for enabling stable and controlled bubble trapping, which is vital for developing bubble-based components like micropumps, micromixers, and microactuators.^228^ These components leverage controlled bubble expansion to facilitate various functionalities, including enhancing mixing efficiency, regulating fluid flow, and deflecting or sorting moving particles within microchannels.^228^ Such capabilities are particularly valuable in microfluidic systems designed for diagnostic applications, where precise fluid manipulation and controlled reagent transport are critical.^229^

The study conducted by Cheng and Gupta^230^ showed the potential of initiated chemical vapor deposition (iCVD) in applying both hydrophilic and hydrophobic coatings onto microfluidic polymeric devices. Although their work focused on coating AM PLA and ABS lattices rather than microfluidic devices, iCVD offers significant advantages for coating complex AM geometries, as it is a solvent-free process that eliminates surface tension effects, enabling conformal coating on intricate structures.^231^ The deposition process is initiated by a heated filament at the top of the coating chamber, which directly drives polymerization.^230^ This mechanism facilitates the controlled deposition of polymer layers, effectively forming the desired coating.^230^

A key factor influencing coating thickness and uniformity in iCVD is the substrate temperature, such that lower temperatures accelerate polymerization rates, leading to thicker coatings.^232^ Consequently, managing the thermal gradient across the substrate is crucial. To maintain low substrate temperatures and promote polymerization, substrates are typically placed on a cooling stage.^233^ However, tall substrates and thermally insulating materials like polymers present additional challenges, as they hinder efficient heat dissipation.^230,234^ This may exacerbate temperature gradients across the substrate, potentially resulting in uneven coating thickness.^230^ Despite that, Cheng and Gupta^230^ reported that optimizing substrate orientation, substrate temperature, and filament temperature, can potentially reduce the thermal gradient effects, highlighting the adaptability of iCVD for engineering both hydrophilic and hydrophobic surfaces on complex AM devices.

#### 3.4.2 Drug Delivery

As discussed in Section 2, AM polymeric devices are gaining prominence in drug delivery due to their customizable designs and tunable material properties, enabling precise drug loading and targeted drug delivery, as well as controlled and sustained drug release.^235^ However, a review of the literature based on the specified keywords described in the Supporting Information reveals a surprising lack of research on leveraging surface engineering to enhance the functionality of AM polymer-based drug delivery systems.

One notable study by Gill et al.^20^ demonstrated the potential of surface engineering to facilitate magnetic targeting in drug delivery systems based on AM microcontainers (MCs). According to this approach, external magnetic fields are used to guide magnetic drug carriers to specific target sites within the body, enabling precise delivery (Figure 16).^236^

**Figure 16:**
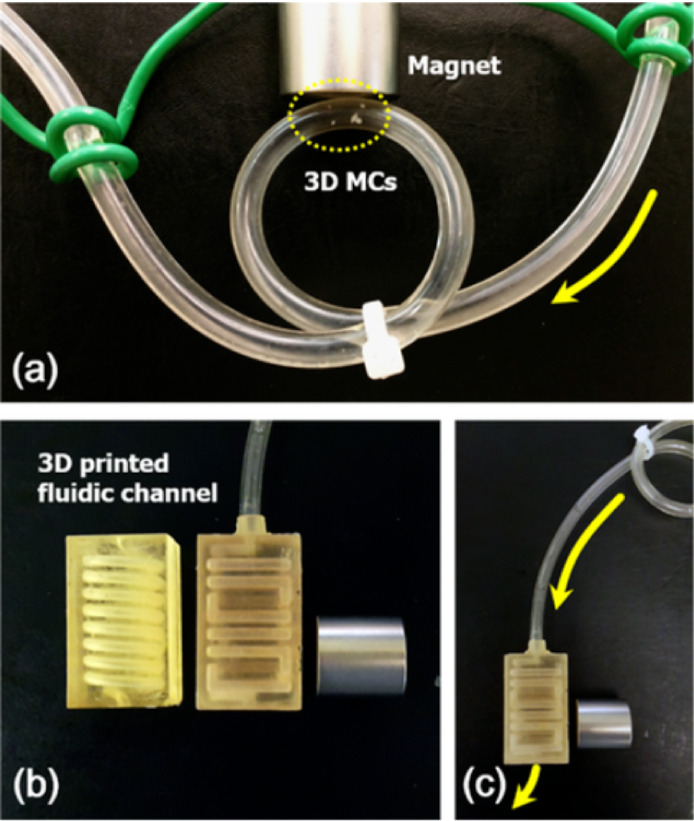
Test setup of Gill et al.^20^ showing the movement of MCs coated with nickel–gold core–shell nanowires (NWs) being guided by a permanent magnet within (a) a tube and (b,c) complex-structured microfluidic. Reproduced with permission from ref 20. Copyright 2017 John Wiley and Sons.

The MCs were fabricated using PolyJet printing with VeroWhite, a photopolymer material.^237^ By precoating the MCs with 3-mercaptopropyltriethoxysilane (3-MPTES), thiol groups were introduced, which allowed for the subsequent attachment of nickel–gold core–shell nanowires (NWs) on the surface.^20^ The ferromagnetic properties of the NW coating facilitated the guided delivery of the MCs within human blood vessel-mimicking channels under the influence of an external magnetic field, as shown in Figure 13.^20^ This innovative approach highlighted the versatility of surface modification techniques in controlling drug movement and release within complex biological environments. Despite this promising finding, knowledge in this field remains limited, and further research is necessary to understand the potential role of surface engineering in enhancing the functionalities of AM polymeric drug delivery systems.

## 4 Limitations and Future Considerations

### 4.1 Search Strategy and Literature Analysis

Several limitations should be acknowledged with the search strategy (as detailed in Supporting Information) that may unintentionally result in omissions of contributions relevant to the scope of this literature review. First, the literature search was conducted exclusively using Scopus, which, while offering broad coverage, does not encompass all emerging journals. Consequently, relevant studies from newer or less widely indexed sources may have been overlooked. Furthermore, while the keywords used in the search strategy were designed to cover a broad range of common terminologies associated with the topic of this research, the inconsistency in terminology across the literature could still lead to missing studies that employed less common or unfamiliar terms. Collectively, these factors may limit the overall comprehensiveness of the review.

Furthermore, the categorization of surface engineering techniques was based on a thematic grouping of the biomedical applications targeted in the reviewed studies. The aim was to highlight the progress made in well-researched areas, such as bone-related applications, to inspire innovative research that will address current gaps in the field. Moreover, the review aimed to emphasize underexplored, yet promising areas related to AM polymeric biomedical scaffolds and devices, such as microfluidics, drug delivery, and tissue engineering beyond bone, with a goal of providing a foundation for future exploration and development. While this approach offers a structured analysis, it may not fully capture the interdisciplinary nature of certain techniques that overlap across multiple biomedical fields.

### 4.2 Long-Term Stability and Sterilization Considerations

Despite the potential of AM polymeric biomedical devices, several challenges must be addressed to improve its feasibility in clinical settings. Beyond issues of reproducibility and defects inherent to AM,^238^ this section focuses on two critical, surface-engineering-related aspects often disregarded in the literature.

First, the complex geometry of AM devices presents significant challenges for surface modification. Achieving uniform surface coatings on porous AM scaffolds, for instance, requires precise optimization of deposition techniques and process parameters to ensure consistent coverage and functionality.^239,240^ Furthermore, the intricate porous structures can lead to the retention of unreacted chemicals (such as cross-linkers and initiators) involved in the surface modification process. These chemicals may become trapped within the scaffold’s pores, and over time, they can be gradually released, potentially causing cytotoxic effects which compromise the long-term stability of the surface modification effects.^241^ Notably, there is a lack of evaluation concerning the stability and longevity of surface engineering effects under physiological conditions. Addressing this gap is crucial to ensuring that the surface modifications—whether coatings, treatments, or physical functionalization, remain safe, stable and effective throughout their intended lifespan in clinical applications.

Second, sterilization is another crucial consideration in the practical adoption of AM biomedical devices. All implanted materials must undergo sterilization to mitigate infection risks at implantation sites.^242,243^ However, little attention has been given to how sterilization processes might affect surface-engineered AM polymeric biomedical devices, particularly those designed for use within the body.

The choice of the sterilization method depends largely on the polymer type. The FDA-established “Category A” sterilization methods, which have a long history of safe and effective use on medical devices include moist heat, dry heat, ethylene oxide (EtO), radiation, and, as of 2024, vaporized hydrogen peroxide (VHP).^244,245^ Among these, EtO is typically used for heat-sensitive polymers like PLA and PCL due to its low operating temperature (37 to 63 °C),^246^ minimizing the degradation risks of these materials.

However, EtO sterilization remains controversial due to its potential to leave toxic residues that can reduce biocompatibility poststerilization.^247,248^ The recent FDA approval of VHP as a sterilization method provides a potential alternative, as it operates at around 50 °C and does not leave toxic residues.^249,250^ However, its material compatibility and inability to penetrate large and dense packaging materials, which may also apply to biomedical devices with porous or multilayered structures, remains a concern that requires further investigation.^251,252^

Importantly, sterilization itself acts as a surface treatment that may interfere with, alter or even degrade surface modifications intended to enhance the functionality of AM polymeric biomedical devices. Given the wide variation in surface engineering approaches, the compatibility between specific sterilization methods and surface-engineered biomedical devices must be carefully evaluated to ensure device safety and performance in clinical applications.

Addressing the challenges related to the stability of surface modifications and the impact of sterilization on surface-engineered devices is a critical step in ensuring their clinical feasibility. These factors directly influence the safety, functionality, and long-term performance of the devices. Once these fundamental issues are resolved, more complex considerations such as regulatory hurdles and standardization can be more effectively tackled to support wider clinical adoption.

### 4.3 Enabling Biomedical Innovation through the Synergy of Polymer, AM, and Surface Engineering

Polymeric materials play a pivotal role in advancing the capabilities of AM in biomedical applications. The versatility and wide availability of polymers, combined with their ease of processing via AM technologies, have broadened the scope of personalized medicine. Polymer AM has facilitated the fabrication of devices for both soft and hard tissue engineering, with high-performance thermoplastics such as PEEK showing great potential for load-bearing implants. Additionally, the polymers’ tunable degradability—an essential feature for tissue regeneration and drug delivery—has made them highly attractive for controlled therapeutic applications. The versatility of polymers extends to microfluidics, where they have enabled the development of innovative *in vitro* diagnostic devices. The ability to functionalize polymers, incorporating bioactive agents or modifying their properties through surface engineering, significantly enhances their therapeutic potential. These innovations have made polymers an important class of material for biomedical applications, providing the flexibility needed for patient-specific biomedical requirements.

However, the distinction between polymeric biomedical devices fabricated through conventional manufacturing methods and those produced via AM is crucial. Conventional manufacturing methods are often limited to simpler geometries, which means surface modifications are typically confined to straightforward structures. In contrast, AM enables the fabrication of intricate and highly customizable geometries through its layer-by-layer deposition process, offering unique opportunities to enhance the performance of polymeric biomedical devices. Unlike conventional approaches, AM unlocks the simultaneous adjustment of three interconnected factors: device design, print parameters, and surface modification strategies, all of which work together to influence the overall functionality of the device.

The flexibility of AM in enabling the fabrication of patient-specific devices is a key factor in expanding the scope of polymeric biomedical applications, from controlling scaffold porosity to creating patient-specific implants. Beyond device design, the ability to simultaneously adjust print parameters and surface modification parameters provides a more holistic approach to enhancing device functionality. For example, as exemplified in Section 3, the ability to modify coating parameters like immersion time and coating concentration alongside print parameters like infill density can impact the coating absorption, ultimately influencing the overall functionality of the device.^37^ Moreover, AM enables innovations such as combining FFF with microcellular foaming to create scaffolds with hierarchical structures that improve bioactivity,^36^ or tailoring the staircase morphology of FFF with specific coating strategies to develop thin-walled structures that provide topological cues that support tissue regeneration.^222^ Additionally, integrating surface treatments like plasma treatment directly into the printing process ensures enhanced coverage and uniformity, as the plasma is applied layer-by-layer.^38^

These innovations are not achievable through conventional methods. Thus, future studies should leverage the interplay between these factors to open new possibilities in the design and functionality of polymeric biomedical devices.

## 5 Conclusions

This systematic review aims to address two key questions: *(1) What surface engineering techniques are applied to polymeric devices produced by additive manufacturing (AM), and (2) how do they enhance the performance of these devices in biomedical applications?*

Our findings reveal that bone tissue engineering dominates the research landscape, with the majority of studies leveraging surface engineering and surface treatments to enhance the osteoinductivity, osteoconductivity, and osseointegration of AM bone scaffolds and implants. Strategies include physical modifications to optimize surface topography, chemical functionalization to introduce bioactive functional groups, and biomimetic approaches incorporating extracellular matrix (ECM)-mimicking components such as minerals, proteins, and growth factors. Beyond increased bioactivity, functional coatings have also been applied to impart antibacterial properties, modulate inflammation, and improve mechanical performance. However, these factors are often overlooked in favor of bioactivity alone, despite their critical role in the overall performance and longevity of bone scaffolds in clinical applications.

While bone tissue engineering remains the primary focus in the literature, surface engineering for AM polymeric biomedical devices has also been explored in vascular, adipose, cartilage, skin, and skeletal muscle regeneration. Similar strategies, including physical and chemical modifications and the incorporation of bioactive and functional materials, have been employed to tailor surfaces to the biological or functional needs of the respective fields. Notably, the research in these fields highlights how AM has enabled innovative hybrid strategies. For instance, combining electrospinning with AM has reinforced vascular scaffold mechanics, fused filament fabrication (FFF) printing with microcellular-foamed filaments has facilitated hierarchical scaffold fabrication with enhanced bioactivity, and sacrificial template-assisted coating has enabled the creation of thin-walled structures with topological cues to guide cellular responses. Collectively, research in these fields has largely proved the adaptability of AM polymeric biomedical devices in both soft and hard tissue applications, where polymer selection and composition tuning play a key role.

Beyond tissue engineering, surface engineering has also demonstrated significant potential in enhancing the functionalities of AM polymer-based microfluidic and drug delivery systems. In microfluidics, surface modifications have been employed to fine-tune the wettability of Lab-on-a-Chip (LOC) devices, improving their applicability in *in vitro* diagnostics by enhancing biomolecule immobilization, reducing nonspecific protein adhesion, and enabling precise fluid manipulation. In drug delivery, ferromagnetic coatings have been applied to facilitate magnetic targeting in AM polymeric systems, allowing more controlled and efficient therapeutic delivery.

Overall, this review provides a comprehensive overview of the current progress in surface engineering for AM polymeric biomedical devices. It highlights key advancements in well-established fields like bone tissue engineering while drawing attention to underexplored yet high-potential areas such as microfluidics and drug delivery. Moreover, we identified critical gaps in the reviewed studies, particularly concerning challenges in sterilization and the long-term stability of surface modifications, which warrant further investigation. Nonetheless, the synergy between polymers, AM, and surface engineering presents a promising avenue for driving innovation and expanding the biomedical applications of AM polymeric devices.

## Supplementary Material



## Data Availability

Data will be made available upon request.

## Notes

Detailed literature search strategy adapted from the Preferred Reporting Items for Systematic reviews and Meta-Analyses (PRISMA) guidelines, defined eligibility criteria for study inclusion, summary table of all eligible studies analyzed in this review (classified as primary and secondary sources), publication trend and demographic analyses (PDF)
