# Supplementary material for: Synergistic Advances in Additive Manufacturing and Surface Engineering for Polymeric Biomedical Devices
Source: ACS Polym Au. 2025 Nov 7;5(6):781–810. doi: 10.1021/acspolymersau.5c00102 (PMC12874162; doi:10.1021/acspolymersau.5c00102)
Supplement: Supplementary file 1 [file lg5c00102_si_001.pdf]

Supporting Information

Synergistic advances in additive manufacturing and surface engineering for polymeric biomedical devices

Wei Juene Chong[1], Antonella Sola[2,*], Yuncang Li[1], Paul F.A. Wright[3], and Cuie Wen[1,**]

[1]*Centre for Additive Manufacturing, School of Engineering, RMIT University, Melbourne, Victoria 3001, Australia*

[2]*Department of Sciences and Methods for Engineering (DISMI), University of Modena and Reggio Emilia, Via Amendola 2, Reggio Emilia, 42122, Italy*

[3]*School of Health and Biomedical Sciences, RMIT University, Bundoora, Victoria 3083, Australia*

*Corresponding author, email: antonella.sola@unimore.it

**Corresponding author, email: cuie.wen@rmit.edu.au

1. Literature search strategy

The five steps that constituted the workflow of this systematic review are summarized in **Figure S1.** This literature review was conducted using the PRISMA statement as a guideline, however, modifications were made where necessary, since the PRISMA approach is primarily intended for clinical studies [1, 2]. To address the abovementioned research questions, a literature survey was conducted in Scopus. This archive was selected because it only indexes contributions that have been reviewed by an independent board of experts in their respective field, while covering a wide research scope [3].

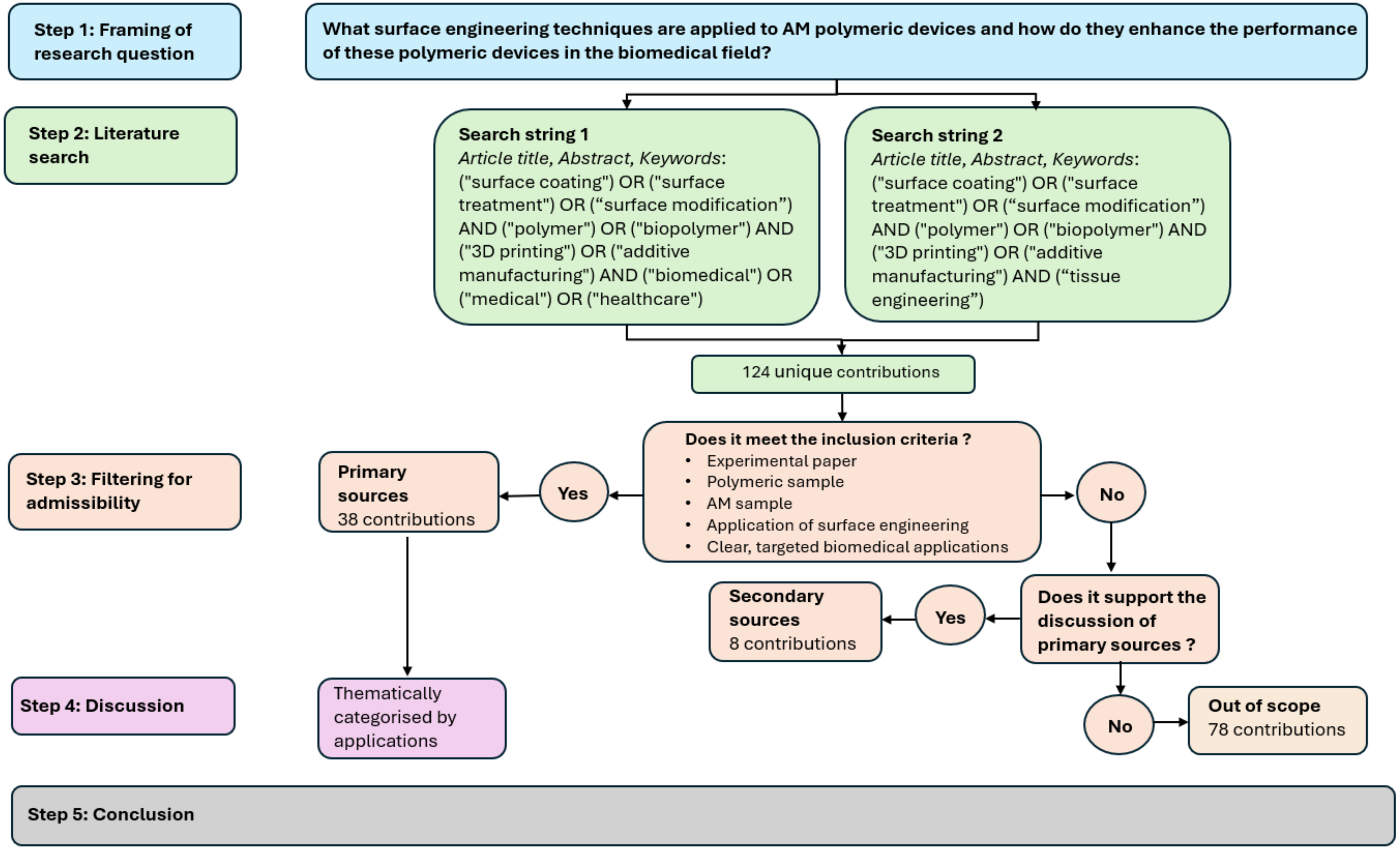

Figure S1. The workflow constituting this systematic review.

The literature survey was conducted on November 27th, 2024, using two search strategies. The first search explored the current trends in surface engineering of AM polymeric devices across the broader field of biomedical applications, while the second focused specifically on tissue engineering applications. This dual search strategy was put in place to ensure a comprehensive coverage on the latest advancements in the field.

The following keywords were used in the two search strategies:

A. *Article title, Abstract, Keywords*:

("surface coating") OR ("surface treatment") OR (“surface modification”)

AND

("polymer") OR ("biopolymer")

AND

("3D printing") OR ("additive manufacturing")

AND

("biomedical") OR ("medical") OR ("healthcare")

B. *Article title, Abstract, Keywords*:

("surface coating") OR ("surface treatment") OR (“surface modification”)

AND

("polymer") OR ("biopolymer")

AND

("3D printing") OR ("additive manufacturing")

AND

("tissue engineering")

Although the literature search was conducted through a combination of the terms ‘surface coating,’ ‘surface treatment,’ and ‘surface modification,’ it is important to clarify the distinction between these terms. Surface coating typically refers to the application of a material, usually a thin continuous layer to a surface, in order to alter its properties [4]. On the other hand, surface treatment and surface modification focus on altering the physical or chemical properties of the surface without necessarily involving the application of a coating [5]. For consistency and to capture the full scope of techniques employed in the literature, this review adopts the term ‘surface engineering’ to include all the various methods used to modify the surface properties of AM polymeric devices, as it encompasses various techniques, including both coating- and non-coating methods.

In addition, the term ‘biopolymer’ was included alongside ‘polymer’ as a search keyword as it is commonly used in a biological context. Similarly, the terms ‘3D printing’ and ‘additive manufacturing’ were both incorporated, as they are often used interchangeably in the literature to describe the same manufacturing processes.

2. Eligibility criteria

The first search string yielded 87 results, and the second returned 55, with 18 duplicates across both searches. After removing the duplicates, 124 unique contributions remained. The abstracts of these contributions were then analyzed, and only experimental papers published in English were further considered for deeper analysis. Among the contributions, 38 experimental studies were deemed eligible for the inclusion as primary sources in this review based on the following criteria:

1. Experimental research
2. Polymeric sample

3. AM sample
4. Application of surface engineering, including surface coating, surface modification, and surface treatment
5. Clear, targeted biomedical applications

Additionally, 8 contributions were classified as secondary sources, which are those that did not directly address the core research questions of this review but were instead cited to support or provide context for findings reported in the primary sources. A summary of the first and secondary sources critically examined for the purpose of this review paper has been included in **Table S1**.

Table S1. Summary of primary and secondary sources.

Author	Year	Title	Country	Polymer	Print technology	Device type	Surface modification	Main effects of surface modification
**Bone-related applications**								
S. Yang, J. Wang, L. Tang, H. Ao, H. Tan, T. Tang, C. Liu	2014	Mesoporous bioactive glass doped-poly (3-hydroxybutyrate-co-3-hydroxyhexanoate) composite Scaffolds with 3-dimensionally hierarchical pore networks for bone regeneration	China	PHBHHx	Micro array printer (extrusion-based)	Scaffold	MBG coating	Enhanced the attachment, activity, and osteogenic differentiation of hMSC.
C.-T.Kao, C.C. Lin, Y.-W. Chen, C.-H. Yeh, H.-Y. Fang, M.-Y. Shie	2015	Poly(dopamine) coating of 3D printed poly(lactic acid) scaffolds for bone tissue engineering	Taiwan	PLA	FFF	Scaffold	PDA coating	Enhanced the adhesion, proliferation and differentiation of hADSC, while demonstrating antibacterial effects against *S. aurues.*
M. Wang, P. Favi, X. Cheng, N.H. Golshan, K.S. Ziemer, M. Keidar, T.J. Webster	2016	Cold atmospheric plasma (CAP) surface nanomodified 3D printed polylactic acid (PLA) scaffolds for bone regeneration	USA; China; Saudi Arabia	PLA	FFF	Scaffold	Cold atmospheric plasma treatment	Enhanced the attachment and proliferation of osteoblast and mesenchymal stem cell.
S.J. Lee et al.	2016	Surface modification of 3D-printed porous scaffolds via mussel-inspired polydopamine and effective immobilization of rhBMP-2 to promote osteogenic differentiation for bone tissue engineering	Korea	PCL	3D bioprinting	Scaffold	PDA/rhBMP-2 coating	Enhanced the proliferation and osteogenic activity of MC3T3-E1.

L.R. Jaidev, K. Chatterjee	2018	Surface functionalisation of 3D printed polymer scaffolds to augment stem cell response	India	PLA	FFF	Scaffold	Alkaline hydrolysis + PEI/CA/calcium deficient HA coating	Enhanced the adhesion, proliferation, and osteogenesis of hMSC.
B.N. Teixeira, P. Aprile, R.H. Mendonça, D.J. Kelly, R.M.d.S.M. Thiré,	2019	Evaluation of bone marrow stem cell response to PLA scaffolds manufactured by 3D printing and coated with polydopamine and type I collagen	Brazil	PLA	FFF	Scaffold	PDA/COL I coating	Enhanced the cellular responses of MSC, with optimal conditions observed during the early stage (< 7 days).
W. Wang, Z. Lu, J. Li, P. Bártolo	2020	Engineering the biological performance of hierarchical nanostructured poly(ε-carpolactone) scaffolds for bone tissue engineering	UK	PCL	3D bioprinting	Scaffold	Acetone immersion + PDA coating	Enhanced the adhesion and spreading of hADSC.
B.I. Oladapo, S.O. Ismail, O.K. Bowoto, F.T. Omigbodun, M. A. Olawumi, M.A. Muhammad	2020	Lattice design and 3D-printing of PEEK with Ca10(OH)(PO4)3 and in-vitro bio-composite for bone implant	UK	PEEK	FFF	Scaffold	HA coating	Enhanced osseointegration, biological activity, and mechanical performance of PEEK scaffold.
E.H. Backes et al.	2021	Development of poly(Ɛ-polycaprolactone)/hydroxyapatite composites for bone tissue regeneration	Brazil	PCL PCL/HA	FFF	Scaffold	Alkaline hydrolysis	Enhanced the cytocompatibility of PCL, with a more significant effect observed for PCL/HA composite scaffolds as compared to PCL.
J.P. Bradford, B. Tucker, G. Hernandez-Moreno, P. Charles, T. Vinoy	2021	Low-temperature inductively coupled plasma as a method to promote biomineralization on 3D printed poly(lactic acid) scaffolds	USA	PLA	FFF	Scaffold	Low temperature plasma treatment	Enhanced PLA scaffold's bioactivity by promoting CaP deposition.
S. Nilawar, K. Chatterjee	2021	Surface decoration of redox-modulating nanoceria on 3D-Printed tissue scaffolds promotes stem cell osteogenesis and attenuates bacterial colonisation	India	PLA	FFF	Scaffold	Alkaline hydrolysis + PEI/CA/CeO_2 coating	Enhanced the osteogenic differentiation of hMSC with reduced ROS levels, as well as improved antibacterial properties.
J. Park, S.J. Lee, T.G. Jung, J.H. Lee, W.D. Kim, J.Y. Lee, S.A. Park	2021	Surface modification of a three-dimensional polycaprolactone scaffold by polydopamine, biomineralisation, and BMP-2 immobilization for potential bone tissue applications	Korea	PCL	3D bioprinting	Scaffold	PDA/HA/BMP-2 coating	PDA/HA coating enabled sustained release of BMP-2 for up to 21 days. The coating enhanced the proliferation and osteogenic differentiation of MC3T3-E1.
J.M. Seok et al.	2021	Enhanced three-dimensional printing scaffold for osteogenesis using a mussel-inspired graphene oxide coating	Korea	PCL	3D bioprinting	Scaffold	PDA/GO coating	PDA enabled controlled deposition of GO, where PDA/GO coating enhanced osteogenesis while remaining non-cytotoxic.
X. Han, N. Sharma , S. Spintzyk, Y. Zhou, Z. Xu, F.	2022	Tailoring the biologic responses of 3D printed PEEK medical implants by plasma functionalization	China; Germany	PEEK	FFF	Disc	Plasma treatment	Enhanced the adhesion, proliferation, and differentiation of SAOS-2, with oxygen plasma contributing to higher bioactivation in comparison to argon plasma.

M. Thieringer, F. Rupp								
E. Filipov et al.	2022	Investigating potential effects of ultra-short laser-textured porous poly-ε-caprolactone scaffolds on bacterial adhesion and bone cell metabolism	Bulgaria	PCL	Extrusion-based printing	Scaffold	Femtosecond laser treatment	Enhanced the attachment, spreading, and proliferation of MG-63 cells, with inhibitory effects observed against S. aureus attachment. Laser-induced microchannels led to greater bioactivity and antibacterial effects as compared to microprotrusions.
S. Iranpour, F. Attari, E. Seyedjafari, J. Nourmohammadi	2022	Coating of 3D printed poly-e-caprolactone scaffolds with silk sericin enhances the osteogenic differentiation of hunman mesenchymal cells	Iran	PCL	FFF	Scaffold	Alkaline hydrolysis or plasma treatment + sericin coating	Enhanced cell adhesion and penetration into interconnected pores, as well as osteogenic differentiation of mesenchymal cells.
C. Garot et al.	2023	3D-printed osteoinductive polymeric scaffolds with optimised architecture to repair a sheep metatarsal critical-size bone defect	France	PLA	FFF	Scaffold	PEI/PLLL/hyaluronic acid/BMP-2 coating	The film showed *in vitro* and *in vivo* biocompatibility. PLA scaffold geometry with cubic pores incorporated with ≈120 µg cm^{-3} BMP-2 dose demonstrated potential in repairing critical-size defects.
S. Sharma, V. Gupta, D. Mudgal	2023	Experimental investigations on polydopamine coated polylactic acid based biomaterial fabricated using 3D printing for orthopedic applications	India	PLA	FFF	Distal ulna bone plates	PDA coating	Improved the mechanical properties of PLA bone plates through optimizing the parameters of FFF printing and PDA coating.
L. Cassari et al.	2023	Strategies for the covalent anchoring of a BMP-2-mimetic peptide to PEEK surface for bone tissue engineering	Italy	PEEK	FFF	Disc	Aoa-x-GBMP1α (AoaBMP) or N3-x-GBMP1α (N3BMP) functionalisation	Both peptide-functionalized PEEK scaffolds demonstrated enhanced HOB cellular activities, with PEEK-N3BMP inducing a higher calcium deposition compared to PEEK-AoaBMP.
R. Donate, R. Paz, Á. Quintana, P. Bordón, M. Monzón	2023	Calcium carbonate coating of 3D-printed PLA scaffolds intended for biomedical applications	Spain	PLA	FFF	Scaffold	$CaCO_3$ coating	Enhanced the mechanical performance of PLA scaffolds and acted as a buffer to minimize abrupt pH changes during PLA degradation attributed to the release of acidic degradation products.
F.C. Menezes, J.M. Scheibel, G.d.S Balbinot, G.M. Miranda, V.C.B Leitune, F.M. Collares, R.M. D. Soares	2024	Bioactive materials-coated polybutylene-adipate-co-terephthalate 3D-printed scaffolds for application in the bone tissues engineering	Brazil	PBAT	FFF	Scaffold	Gelatin/bioglass or bioglass/gelatin, or HA/gelatin coating	Bioglass was essential for enhancing the scaffold's bioactivity, while HA was crucial for improving its mechanical performance. Bioglass/HA coating which balances bioactivity and mechanical performance thus emerged as the optimal coating for PBAT scaffold.
R. Niveditha, R. Saranya, T.U.	2022	Comparative study on structural enhancement of polymer based medical devices using additive	India	PLA	FFF	Bar	Nickel coating	Enhanced the mechanical performance of PLA bars.

Vishnu, K. Chamundeswari		manufacturing technology and pop (Plating on Plastics)						
M. Schneider, C. Günter, A. Taubert	2017	Co-deposition of a hydrogel/calcium phosphate hybrid layer on 3D printed poly(lactic acid) scaffolds via dip coating: Towards automated biomaterials fabrication	Germany	PLA	FFF	Scaffold	CaP/gelatin or CaP/chitosan coating	Both gelatin and chitosan significantly enhanced the deposition of CaP layer onto PLA scaffolds, demonstrating potential in controlling the synthesis of biomimetic coatings on polymeric biomaterials.
E. Filipov et al.	2024	Surface functionalization of 3D printed poly-ϵ-caprolactone by ultrashort laser mirostructuring and ZnO nanolayer deposition	Bulgaria	PCL	Pneumatric extrusion-based printing	Scaffold	Femtosecond laser treatment + ZnO ALD	ALD was successfully used to coat PCL scaffolds with ZnO layers using varying ALD cycles at 50°C, below the typical ALD processing temperature. Additionally, laser-induced microchannels facilitated higher ZnO adsorption compared to non-laser-treated surfaces.
A. Alhotan, S. Alhijji, S.A. Abdalbary, R.E. Bayoumi, J.P. Matinlinna, T.M. Hamdy, R.M. Abdelraouf	2024	An advanced surface treatment technique for coating three-dimensional-printed polyamide 12 by hydroxyapatite	Saudi Arabia; Egypt	PA 12	Selective laser sintering	Disc	HA coating	The study demonstrated that 10-MDP and light polymerization effectively coated PA 12 with a stable layer of HA.
C.-Y. Liao, P.-L. Wu, C.-Y. Lee	2019	Customized PEEK implants with microporous and surface modification using 3D printing	Taiwan	PEEK	FFF	Disc	HA coating	The study highlighted the potential of cold spraying to coat PEEK with HA layer. However, further optimization of cold spray parameters is necessary to improve coating adhesion to the PEEK surface.
**Vascular-related applications**								
S.J. Lee et al.	2019	Vascular endothelial growth factor immobilised on mussel-inspired three-dimensional bilayered scaffold for artificial vascular graft application: *In vitro* and *in vivo* evaluations	Korea	PCL	Electrospinning + 3D bioprinting	Electrospun tubular scaffold coated with 3D printed PCL strands	PDA/VEGF coating	The study demonstrated the potential of electropsun tubular scaffolds coated with 3D printed PCL strands as mechanically robust vascular scaffolds to support vascular tissue engineering. Additionally, PDA/VEGF coating significantly enhanced vascular cell proliferation and angiogenic differentiation.
J. Kim, S.A. Park, J. Lee	2019	Fabrication and characterization of bioresorbable drug-coated porous scaffolds for vascular tissue engineering	Korea	PCL	3D bioprinting	Scaffold	Plasma treatment + aspirin and atorvastatin calcium salt coating	The study demonstrated the potential of employing drug-coated PCL scaffolds to lower LDL cholesterol and reduce restenosis.
**Adipose-related applications**								
S. Jain, M.A. Yassin, T. Fuoco, H. Liu, S. Mohamed-Ahmed, K. Mustafa, A. Finne-Wistrand	2020	Engineering 3D degradable, pliable scaffolds toward adipose tissue regeneration; optimized printability, simulations and surface modification	Sweden	PLATMC	Pneumatic extrusion	Scaffold	PDA coating	The study highlighted the interplay between print parameters and scaffold's design in influencing the scaffold's mechanical properties. Additionally, PDA coating enhanced stem cell proliferation and adipogenic differentiation.

**Cartilage-related applications**								
X. Deng et al.	2021	Precision 3D printed meniscus scaffolds to facilitate hMSCs proliferation and chondrogenic differentiation for tissue regeneration	China	PU	Cyro-printing (extrusion-based)	Scaffold	COL I or FN coating	Enhanced stem cell functions and long-term chondrogenesis on PU scaffolds, with FN outperforming COL I coating.
S. Zhang et al.	2022	3D-printed polyurethane tissue-engineering scaffold with hierarchical microcellular foam structure and antibacterial Properties	China	TPU	FFF	Scaffold	CO_2 microcellular foaming + PDA/GO coating	FFF printing of microcellular foamed filaments enabled the fabrication of hierarchical scaffolds with interconnected open pores which promoted cell adhesion. Additionally, the PDA/GO coating improved the scaffold's antibacterial properties.
**Skin-related applications**								
N. Khoshnood, A. Zamanian, M. Abbasi	2021	The potential impact of polyethylenimine on biological behavior of 3D-printed alginate scaffolds	Iran	Alginate	3D bioprinting	Scaffold	PEI coating	PEI coating promoted the adhesion, proliferation, and spreading of fibroblasts, while also reducing the degradation rate of alginate scaffolds.
**Skeletal-muscle-related applications**								
S. Miao, M. Nowicki, H. Cui, S.-J. Lee, X. Zhou, D.K. Mills, L.G. Zhang	2019	4D anisotropic skeletal muscle tissue constructs fabricated by staircase effect strategy	USA	PVA	FFF	Sacrificia l template	PCL and shape memory polymer coating	The study demonstrated the potential of sacrificial template-assisted coating to create thin-walled structures by firstly coating the printed sacrificial template with a polymer solution, followed by template dissolution. The resulting structure featured topographical cues from the staircase effect of FFF, guiding skeletal muscle tissue regeneration. Additionally, the use of shape-memory polymer solution as the coating showed promise for creating thin-walled structures with shape-memory properties.
**Microfluidics**								
P. Pokharna, M.K. Ghantasala, E.A. Rozhkova	2018	Surface modification and characterization of polylactic acid (PLA) 3D printed structures for cell culture applications	USA	PLA	unspecified	LOC device	Hydrolysis or UV ozone plasma treatment or gold-film coating	Surface modification via hydrolysis emerged as the most efficient and optimum method to enhance protein attachment on the surface of PLA-based LOC device as compared to UV ozone plasma and gold-film coating.
P. Pokharna, M.K. Ghantasala, E.A. Rozhkova	2021	3D printed polylactic acid and acrylonitrile butadiene styrene fluidic structures for biological applications: Tailoring bio-material interface via surface modification	USA	PLA and ABS	FFF	Scaffold	Hydrolysis or UV ozone plasma treatment or gold-film coating	Surface modification via hydrolysis emerged as the most efficient and optimum method to enhance protein attachment on the surface of PLA- and ABS-based LOC device as compared to UZ ozone plasma and gold-film coating.
C. Cheng, M. Gupta	2017	Surface functionalization of 3D-printed plastics via initiated chemical vapor deposition	USA	PLA and ABS	As received printed parts	Lattice	Hydrophobic PPFDA and hydrophilic P(HEMA-co-EGDA) coating	The study demonstrated the potential of utilizing iCVD to coat thermally insulating PLA and ABS substrates with both hydrophobic and hydrophilic coatings through optimizing substrate orientation and substrate temperature.

[15] L. Brandhoff, S. van den Driesche, F. Lucklum, M.J. Vellekoop	2015	Creation of hydrophilic microfluidic devices for biomedical application through stereolithography	Germany	Photopolymer resin	SLA printed	Test device	PEG coating	The study showed the potential for PEG coating to enhance surface hydrophilicity to minimize non-specific protein adhesion during ELISA.
**Drug delivery**								
W.A. Gill et al.	2017	Functionalization of 3D printed micro-containers with Ni-Au core-shell nanowires	Korea; UAE	Photopolymer resin	Polyjet printing	Micro-container	3-MPTES + Ni-Au core-shell NWs coating	The coating showed promise in facilitating magnetic-targeted delivery of VeroWhilte micro-containers.
**Secondary sources**								
**Author**	**Year**	**Title**	**Country**	**Polymer**	**Print technology**	**Device type**	**Surface modification**	**Main findings**
N.E. moataz bellah Ahmed, A.A. el azeem, F.S. Bayoumi	2018	The effect of 3D-printed polycaprolactone (PCL) scaffold with different surface coatings on the immunogenicity of stem cells from human exfoliated deciduous teeth (SHED)	Egypt	PCL	FFF	Scaffold	HA and multi-walled carbon nanotube (MWCNT) coating	The study explored the impact of commonly used bioactive and functional materials on the immunogenicity of mesenchymal stem cells, emphasizing the importance of considering these coatings' effects to prevent immune rejection in regenerative applications.
X. Han et al.	2019	Carbon fiber reinforced PEEK composites based on 3D-printing technology for orthopedic and dental applications	Germany, China	PEEK and carbon-reinforced PEEK	FFF	Disc	Mechanical polishing and sandblasting	The study explored the impact of polishing on FFF-printed structures. The results showed higher cell densities on unpolished surfaces due to increased surface roughness, compared to the smooth, polished surfaces. Additionally, carbon fiber reinforcement slightly improved the compressive strength of PEEK, demonstrating its potential as a bone graft material for tissue engineering.
F. Liu, W. Wang, W. Mirihanage, S. Hinduja, P.J. Bartolo	2018	A plasma-assisted bioextrusion system for tissue engineering	UK	PCL	Customised printer plasma + extrusion	Scaffold	Plasma treatment	The study introduced an innovative approach that integrates plasma treatment into the layer-by-layer printing process, allowing for plasma treatment to permeate the entire printed structure, including the interconnected regions. The results demonstrated that plasma-treated scaffolds significantly enhanced biological performance.
S. Mohsenimehr, M.R. Khani, N. Fani, M.R.B. Eslaminejad, B. Shokri, A. Ghassami	2020	Surface modification of PLA scaffold using radio frequency (RF) nitrogen plasma in tissue engineering application	Iran	PLA	FFF	Scaffold	Plasma treatment	The study explored the effects of the type and composition of the gas (N_2, N_2/O_2, N_2/H_2) utilized for plasma treatment on the bioactivity of PLA. The results showed that N_2/O_2 (1:1) plasma treated group demonstrated the best improvement in biocompatibility.

P. Kowalczyk, P. Trzaskowska, I. Łojszczyk, R. Podgórski, T. Ciach	2019	Production of 3D printed polylactide scaffolds with surface grafted hydrogel coatings	Poland	PLA	FFF	Disc and scaffold	Polyvinylpyrrolidone (PVP) coating	The study emphasized the influence of substrate geometry on the toxicity of PVP coatings. Cytotoxicity was observed in porous scaffolds, as their structure allowed for deeper penetration and retention of chemicals during the grafting process. These chemicals were gradually released, leading to cytotoxic effects. Additionally, the impact of PVP molecular weight on coating thickness and uniformity was explored, with higher molecular weights resulting in uneven coating distribution.
S. Bertlein, G. Hochleitner, M. Schmitz, J. Tessmar, M. Raghunath, P.D Dalton, J. Groll	2019	Permanent hydrophilization and generic bioactivation of melt electrowritten scaffolds	Germany	PCL	MEW	Scaffold	Six-arm star-shaped NCO-poly(ethylene oxide-stat-propylene oxide) (sP(EO-stat-PO)) hydrogel coating	The study demonstrated the potential of sP(EO-stat-PO) coatings in preventing non-specific protein adsorption onto the scaffold, which can help reduce inflammation and enhance biocompatibility.
I. Aktitiz, F. Darıcık, A. Aydın, K. Aydın	2024	Metallization of 3D printed polylactic acid polymer structures via radio-frequency sputtering	Turkey	PLA	FFF	Cube	Aluminum coating	The study demonstrated the potential for aluminium coating to reduce the degradation of PLA.
R. Donate, M.E. Alemán-Domínguez, M. Monzón	2021	On the effectiveness of oxygen plasma and alkali surface treatments to modify the properties of polylactic acid scaffolds	Spain	PLA	FFF	Scaffold	Alkaline hydrolysis or plasma treatment	The study compared the effects of alkaline hydrolysis and plasma treatment on surface hydrophilicity and the enzymatic degradation rate of PLA. The results indicated that hydrolysis resulted in higher PLA degradation rates, while plasma treatment increased surface hydrophilicity with minimal impact on bulk properties and mechanical strength. However, a limitation of plasma treatment is the non-permanent nature of the hydrophilicity it induces.

3. Statistics and demography

As shown in **Figure S2**, the interest in surface engineering for AM polymeric devices in biomedical applications first emerged in 2014 and steadily increased up to 2019. Although a decline was observed in 2020, interest peaked again in 2021. Following a slight decline in 2022, the interest has remained stable up till 2024. The peak observed in 2021 could be attributed the heightened focus on biomedical research prompted by the Covid-19 crisis [6, 7].

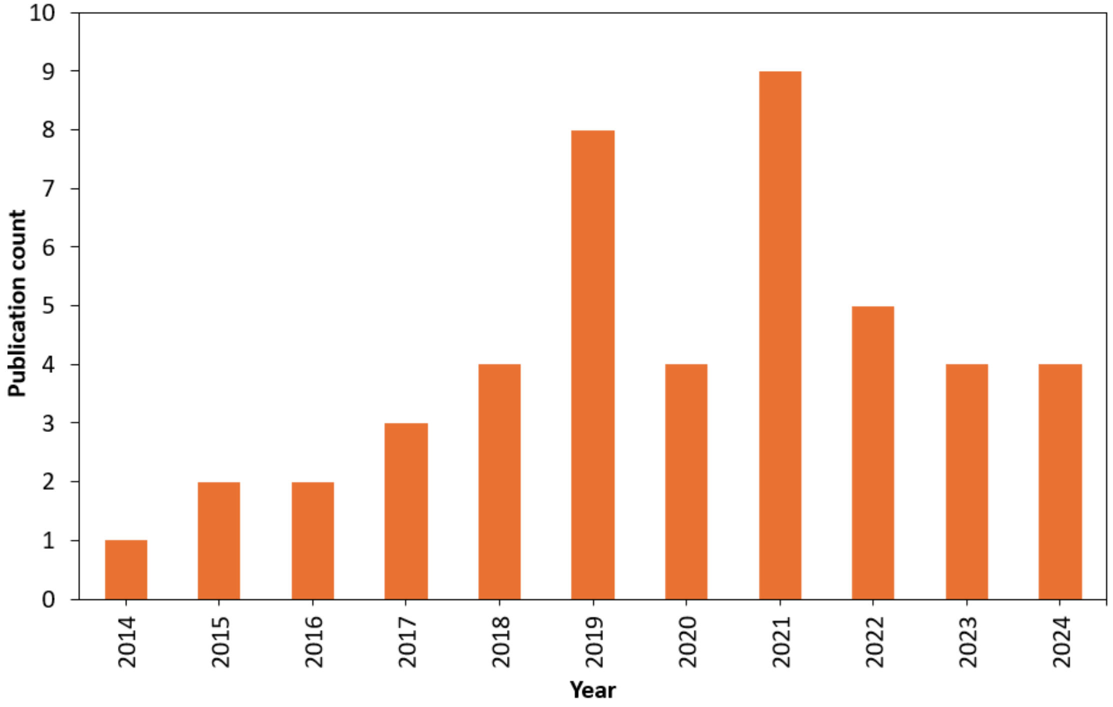

Figure S2. Publication counts by year between 2014 to 2024.

The overall increase in research activity recorded over the last 11 years is likely driven by the advancements in AM, particularly the wider availability and affordability of fused filament fabrication (FFF, a.k.a fused deposition modeling, FDM), for commercial applications following the expiry of Stratasys' patent in 2009 [8, 9]. Since FFF primarily works with polymers [10], it is reasonable to expect a concurrent rise in the interest of polymeric AM. Notably, more than 50% of the reviewed studies employed FFF as their AM technology, while the remaining studies utilized alternative AM methods, as shown in **Figure S3**. In terms of the polymers investigated, polylactic acid (PLA) accounted for the largest portion of studies (37%), followed by polycaprolactone (PCL) (28%), polyether ether ketone (PEEK) (11%), and other polymers comprising the remaining 24% (**Figure S4**).

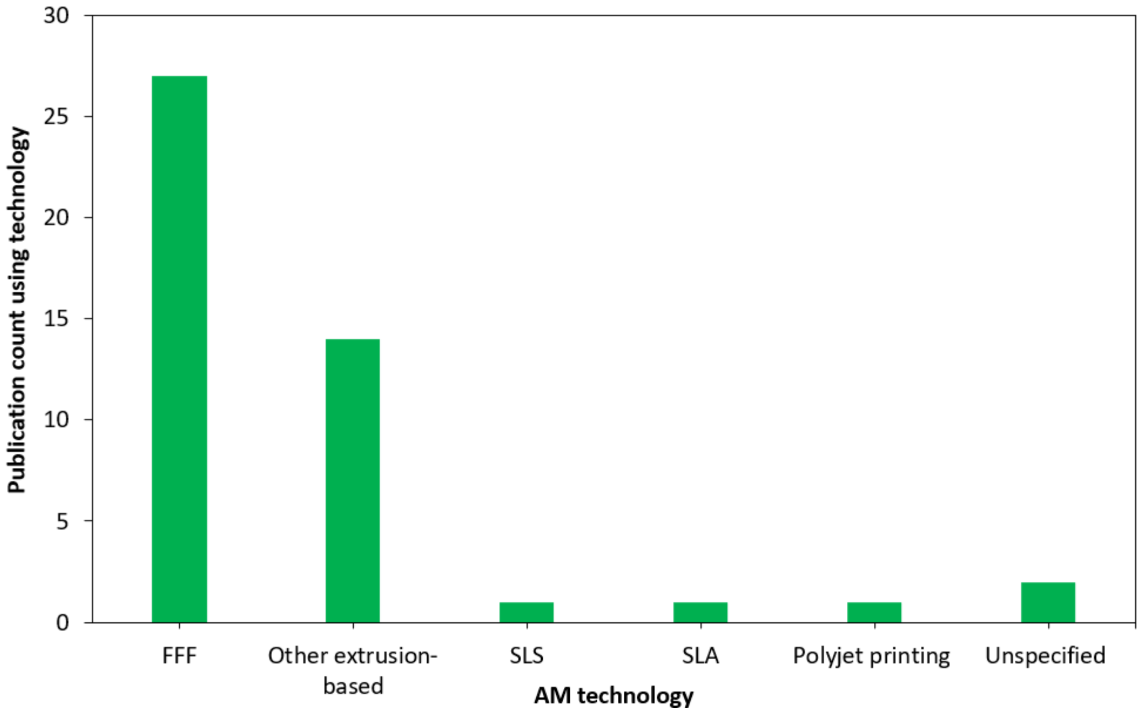

Figure S3. Publication counts for the different AM technologies utilized. Technologies are reported using their commonly used acronyms and in brackets as defined by ISO/ASTM 52900:2021 [11]. FFF – Fused Filament Fabrication (Material Extrusion, MEX); SLS – Selective Laser Sintering (Powder Bed Fusion, PBF); SLA – Stereolithography (Vat Photopolymerization VP); Polete printing (Material Jetting, MJ).

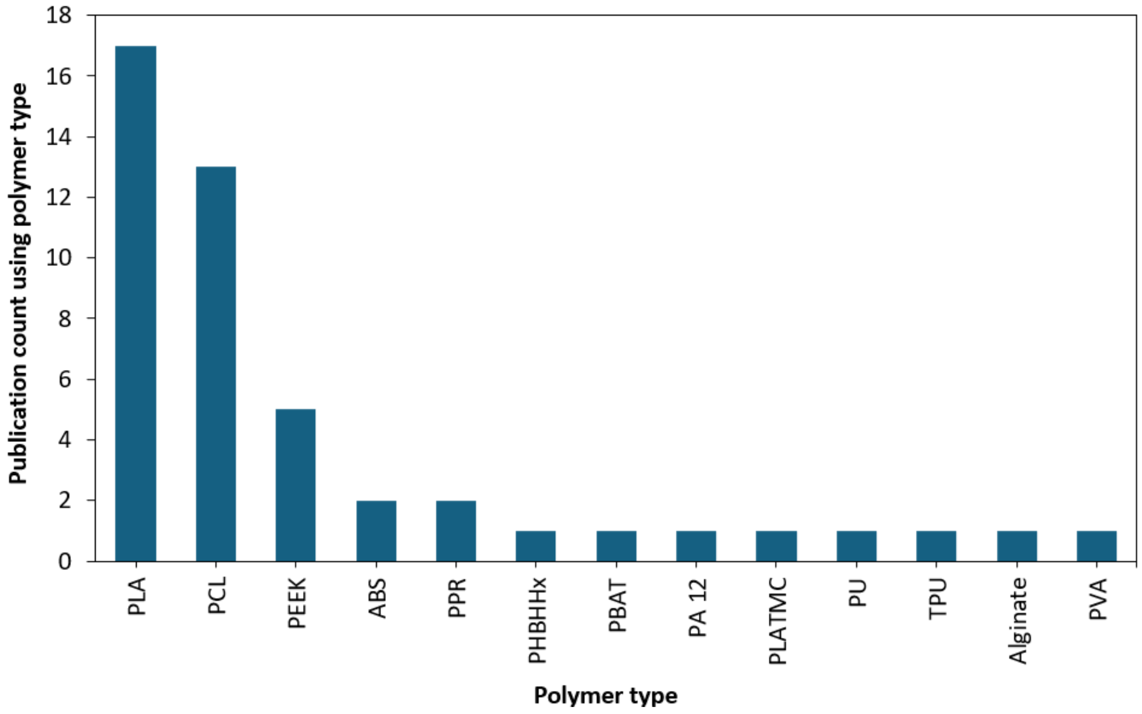

Figure S4. Publication counts for the different polymer utilized: PLA - Polylactic acid, PCL - Polycaprolactone, PEEK - Polyether ether ketone, ABS - Acrylonitrile butadiene styrene, PPR - Photopolymer resin, PHBHHx - poly(3-hydroxybutyrate-co-3-hydroxyhexanoate), PBAT - Poly(butylene adipate-co-terephthalate), PA 12 - Polyamide 12, PLATMC - Polylactic acid trimethylene carbonate, PU - Polyurethane, TPU - Thermoplastic polyurethane, PVA - Polyvinyl alcohol. Note: The total number of contributions in the figure exceed 38, as some studies compared two or more polymers, leading to multiple counts for a single contribution.

Based on the demographic analysis of the first and corresponding authors of the reviewed studies (**Figure S5**), the research on surface engineering of polymeric biomedical devices has received global attention, with the highest levels of interest observed across Asia and Europe, each corresponding to 30% of the total studies. When considering individual countries, South Korea, India, USA, China, and Germany, have emerged as leading contributors to this field, each accounting for at least 10% of the total studies, including those involving international collaborations. This trend is not surprising, given that both USA and Europe are home to key polymeric AM players, such as Stratasys, Ultimaker, and Prusa. Moreover, Asia has seen rapid growth in AM markets in recent years [12], thus, driving significant research efforts into AM.

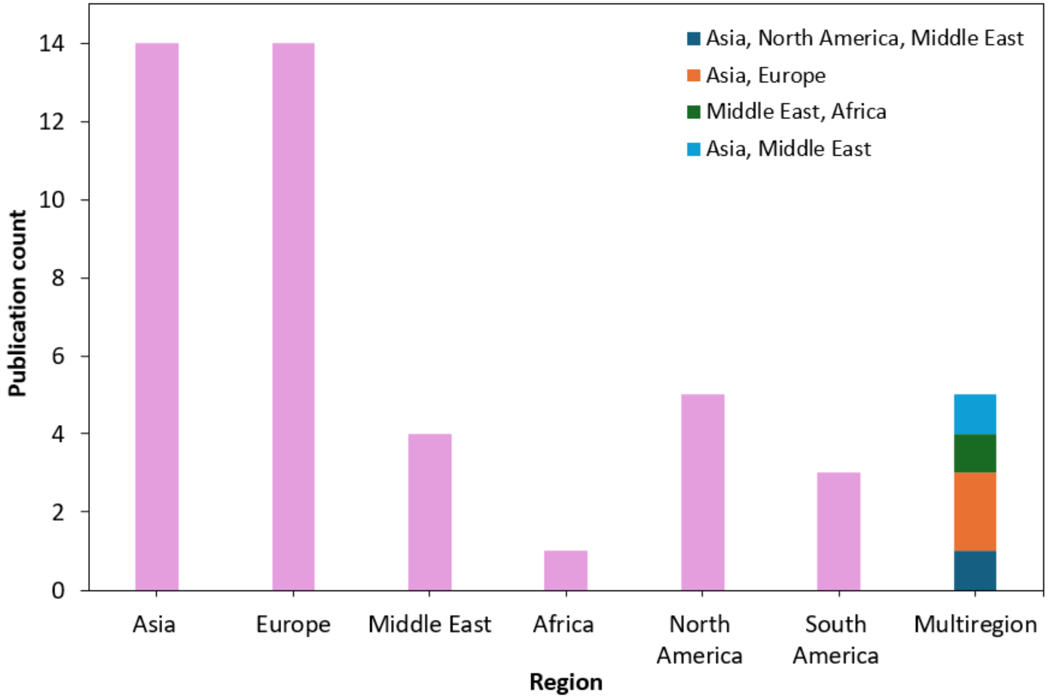

Figure S5. Publication counts by region.

Furthermore, the literature search brought to light some thematic categories within the broader biomedical applications of surface-engineered AM polymeric devices. Tissue engineering was identified as the predominant targeted application, with a prevalent attention towards bone tissue engineering. A smaller portion of studies explored other applications beyond tissue engineering, particularly, microfluidics and drug delivery. According to the Stem Cell Council [13], several of the leading contributing countries mentioned above, including USA, South Korea, Germany, and China, have been listed as pioneers in the research and development of tissue regenerative medicine, further reinforcing their position as primary contributors to the field.

Importantly, the progress in polymer AM has played a key role in driving innovation in surface engineering, as the capability of AM to fabricate complex, patient-specific structures combined with the versatility of polymers, has significantly influenced the development of biomedical applications [14, 15].

Supplementary references

[1] M.J. Page, J.E. McKenzie, P.M. Bossuyt, I. Boutron, T.C. Hoffmann, C.D. Mulrow, L. Shamseer, J.M. Tetzlaff, E.A. Akl, S.E. Brennan, R. Chou, J. Glanville, J.M. Grimshaw, A. Hróbjartsson, M.M. Lalu, T. Li, E.W. Loder, E. Mayo-Wilson, S. McDonald, L.A. McGuinness, L.A. Stewart, J. Thomas, A.C. Tricco, V.A. Welch, P. Whiting, D. Moher, The PRISMA 2020 statement: An updated guideline for reporting systematic reviews, Br. Med. J. 372 (2021) n71.

[2] M.J. Page, D. Moher, P.M. Bossuyt, I. Boutron, T.C. Hoffmann, C.D. Mulrow, L. Shamseer, J.M. Tetzlaff, E.A. Akl, S.E. Brennan, R.Chou, J. Glanville, J.M. Grimshaw, A. Hróbjartsson, M.M Lalu, T. Li, E.W. Loder, E. Mayo-Wilson, S. McDonald, L.A. McGuinness, L.A. Stewart, J. Thomas, A.C. Tricco, V.A. Welch, P. Whiting, J.E. McKenzie, PRISMA 2020 explanation and elaboration: updated guidance and exemplars for reporting systematic reviews, Br. Med. J. 372 (2021) n160.

[3] Elsevier, Content policy and selection. https://www.elsevier.com/products/scopus/content/content-policy-and-selection, (Accessed April 4, 2025).

[4] R. Lambourne, R, Paint composition and applications - a general introduction, in: R. Lambourne, T.A. Strivens (Eds.), Paint and surface coatings (second edition), Woodhead Publishing, 1999, pp. 1-18.

[5] S. Bose, S.F. Robertson, A. Bandyopadhyay, Surface modification of biomaterials and biomedical devices using additive manufacturing, Acta Biomater. 66 (2018) 6-22.

[6] A. Shafiee, L. Moradi, M. Lim, J. Brown, Coronavirus disease 2019: A tissue engineering and regenerative medicine perspective, Stem Cells Transl. Med. 10 (2021) 27-38.

[7] C. Sohrabi, G. Mathew, T. Franchi, A. Kerwan, M. Griffin, J.S.C.D Mundo, S.A. Ali, M. Agha, R. Agha, Impact of the coronavirus (COVID-19) pandemic on scientific research and implications for clinical academic training – A review, Int. J. Surg. 86 (2021) 57-63.

[8] J. Kajbič, G. Fajdiga, J. Klemenc, Material extrusion 3D printing of biodegradable composites reinforced with continuous flax fibers, J. Mater. Res. Technol. 27 (2023) 3610-3620.

[9] A. Sola, R. Rosa, A.M. Ferrari, Environmental impact of fused filament fabrication: What is known from life cycle assessment?, Polym. 16 (2024) 1986.

[10] E. Cuan-Urquizo, E. Barocio, V. Tejada-Ortigoza, R.B. Pipes, C.A. Rodriguez, A. Roman-Flores, A characterization of the mechanical properties of FFF structures and materials: A review on the experimental, computational and theoretical approaches, Mater. 12 (2019) 89562.

[11] Additive manufacturing - General principles - Fundamentals and vocabulary, ISO/ASTM 52900, Nov. 2021. [Online]. Available: https://www.iso.org/standard/74514.html.

[12] AMFG, Additive manufacturing around the world: What is the state of 3d printing adoption in north america and europe?. https://amfg.ai/2019/11/07/additive-manufacturing-around-the-world-what-is-the-state-of-3d-printing-adoption-in-north-america-and-europe/, (Accessed Apr 4, 2025).

[13] Stem Cell Council, Best countries in the world for regenerative medicine. https://www.stemcellcouncil.com/article/best-countries-in-the-world-for-regenerative-medicine, (Accessed Apr 5, 2025).
[14] A.B. Singh, Transforming healthcare: A review of additive manufacturing applications in the healthcare sector, Eng. Proc. 72 (2024) 2.
[15] C. Li, D. Pisignano, Y. Zhao, J. Xue, Advances in medical applications of additive manufacturing, Eng. 6 (2020) 1222-1231.
